# AdMSC-derived exosomes alleviate acute lung injury via transferring mitochondrial component to improve homeostasis of alveolar macrophages

**DOI:** 10.7150/thno.69533

**Published:** 2022-03-21

**Authors:** Liangjun Xia, Chunli Zhang, Nianyin Lv, Zihao Liang, Tonghui Ma, Haibo Cheng, Youbing Xia, Liyun Shi

**Affiliations:** 1 Key Laboratory of Inflammation and Immunoregulation, School of Medicine and Holistic Integrative Medicine, Nanjing University of Chinese Medicine, Nanjing City 210046, China; 2 Department of Clinical Research, Friendship Plastic Surgery Hospital, Nanjing Medical University, Nanjing City 210029, China; 3 School of Medicine and Holistic Integrative Medicine, Nanjing University of Chinese Medicine, Nanjing City 210046, China; 4 The First School of Clinical Medicine, Affiliated Hospital of Nanjing University of Chinese Medicine, Nanjing City 210029, China; 5 Affiliated Hospital of Xuzhou Medical University, Xuzhou Medical University, Xuzhou City 221004, China; 6 Acupuncture and Tuina College, Nanjing University of Chinese Medicine, Nanjing City 210023, China; 7 Shulan International Medical College, Zhejiang Shuren University, Hangzhou City 310022, China

**Keywords:** stem cells, exosomes, acute lung injury, alveolar macrophages, mitochondrial function

## Abstract

**Rationale:** Aberrant activation of macrophages with mitochondria dismiss was proved to be associated with pathogenesis of ALI (acute lung injury). Exosomes from adipose-derived mesenchymal stem cells (AdMSC-Exos) have been distinguished by their low immunogenicity, lack of tumorigenicity, and high clinical safety, but their role in treating ALI and the mechanism involved need to be defined. In this study, we sought to investigate whether the mitochondrial donation from AdMSC-Exos provides profound protection against LPS-induced ALI in mice, accompanied by improvement of macrophage mitochondrial function.

**Methods:** C57BL/6 mice were orotracheally instilled with LPS (1 mg/kg). AdMSC-Exos were administered via the tail vein 4 h after LPS inhalation. Flow cytometry, H&E, Quantitative Real-Time PCR, immunofluorescence (IF), confocal microscopy imaging was conducted to investigate lung tissue inflammation and macrophage mitochondrial function. And further observe the transfer of exosomes and the effect on mitochondrial function of MH-S cells through *in vitro* experiments.

**Results:** AdMSC-Exos can transfer the stem cell-derived mitochondria components to alveolar macrophages in a dose-dependent manner. Likely through complementing the damaged mitochondria, AdMSC-Exos exhibited the ability to elevate the level of mtDNA, mitochondrial membrane potential (MMP), OXPHOS activity and ATP generation, while reliving mROS stress in LPS-challenged macrophages. Restoring mitochondrial integrity via AdMSC-Exos treatment enabled macrophages shifting to anti-inflammatory phenotype, as featured with the down-regulation of IL-1β, TNF-α and iNOS secretion and increase in production of anti-inflammatory cytokines IL-10 and Arg-1. As we depleted alveolar macrophages using clodronate liposomes, the protective role for AdMSC-Exos was largely abrogated.

**Conclusions:** AdMSC-Exos can effectively donate mitochondria component improved macrophages mitochondrial integrity and oxidative phosphorylation level, leading to the resumption of metabolic and immune homeostasis of airway macrophages and mitigating lung inflammatory pathology.

## Introduction

Acute lung injury (ALI) is a clinical syndrome caused by various pathogenic factors, including acute pneumonia, sepsis, severe trauma, and acute pancreatitis. This syndrome is initially characterized by pulmonary inflammation and microvascular permeability, and eventually develops into acute respiratory distress syndrome 1. Despite the improved outcome with mechanical ventilation, the mortality of ARDS still remains as high as 35-55% 2, 3. Lacking effective treatment further deteriorates the condition 4, 5. Infectious agents such as lipopolysaccharide (LPS), the main component of the cell wall of gram-negative bacteria, have been confirmed to induce ALI 6. After entering the body, LPS causes lung damage by primarily sensed by innate immune cells and initiating the secretion of inflammatory mediators. This process leads to the destruction of the alveolar epithelial and endothelial barriers, compromising normal respiratory function of the hosts 7. Thus, it is critical to develop treatments to alleviate lung inflammation and tissue damage.

Mesenchymal stem cells (MSCs) have been proposed to modulate immune responses and inflammation 8, and therefore increasingly recognized as a potential therapeutics for ARDS 9. MSCs from various tissues like adipose tissue demonstrate to improve survival, reduce inflammation, and enhance bacterial clearance in preclinical lung injury models 10-15. However, there exist unsolved issues such a medical safety, ethics and sources that preclude the clinical application of MSC-based treatment. Compared with stem cells themselves, exosomes from MSCs display multiple advantages including low immunogenicity, lack of tumorigenicity, high clinical safety and minimal ethical concerns, making them a potential cell-free therapy for inflammatory diseases 16, 17. Studies have shown that exosomes contain varied compositions including proteins, mRNAs, microRNAs (miRNAs) and lipids, which can be transferred to target cells through ligand/receptor interaction, membrane fusion, and cargo internalization 18, 19.

Mitochondria are known as an energy-producing factory supporting cellular activities through generating ATP. It also serves as a signaling hub and controls a wide range of biological processes such as cellular proliferation, apoptosis, metabolism, immune, etc. The mitochondrial respiratory chain is essential for electron transport and energy conversion, disruption of which would affect energy metabolism and cause loss of tissue homeostasis. Evidences have shown that dysfunctional mitochondria would disturb metabolic fitness of alveolar epithelial cells and macrophages, contributing to a variety of lung diseases, such as ARDS, COPD, lung cancer, asthma, pulmonary arterial hypertension or idiopathic pulmonary fibrosis 20, 21. Clinically, mitochondrial dysfunction was evidenced to be related with higher mortality in septic patients 22. Conversely, survivors of multiple organ dysfunction syndromes demonstrated to have better mitochondrial function with preservation of ATP and biogenesis markers 23. Those data support the importance of mitochondrial integrity in maintaining tissue homeostasis and preventing severe lung disease.

Alveolar macrophages (AMs), positioned in the lumens of airway spaces closed to lung epithelium, are specialized in clearing cellular debris and inflammation resolving. Dysfunctional AMs with abnormal mitochondrial activity are thought to underlie critical lung inflammation 24. Recent data supported that MSC and MSC-derived EVs can potentially reduce lung injury and inflammation, but how MSC-derived EVs regulates pulmonary innate immune cells, particularly AMs, is yet to be explored 25-27. It is currently recognized that cellular metabolic programs, in addition to generating ATP and intermediates for cell biosynthesis, also exert the regulatory effect on cellular signaling pathways, epigenetic signature and hence cellular identity and functionality 28. In macrophages, LPS stimulation was found to induce cellular metabolic conversion to glycolytic metabolism, leading to myeloid cell infiltration and proinflammatory responses 29, 30. By contrast, type 2 cytokines IL-4 and IL-13 induced higher level of mitochondrial oxidative phosphorylation (OXPHOS), and production of tissue repair factors in macrophages 31, 32. These findings may incite an interesting question whether stem cell-derived exosomes, through regulating metabolic programs in macrophages, mediate their regulation on lung inflammation and injury.

Our current study aims to analyze the role of AdMSC-Exos in reducing endotoxin-induced ARDS, and investigate the mechanism involved. The study demonstrates that through transferring mitochondrial component, AdMSC-Exos improve the integrity and function of mitochondria in AMs and promote their shift to an anti-inflammatory phenotype, leading to the lessened ALI.

## Methods

### Experimental animals

Wild-type mice of the C57BL/6 genetic background (8 weeks old) were obtained from a specific pathogen-free (SPF)-level facility (Vital River Laboratories, Beijing, China). They were kept under a controlled 12:12-h light-dark cycle, with a standard diet and free access to water in the Animal Experimental Center of Nanjing University of Traditional Chinese Medicine. The study protocol was approved by the Animal Care and Use Committee of Nanjing University of Chinese Medicine on 6 May 2019 (approval no. 201905A001).

### *In vivo* LPS-induced lung injury model

Pathogen free, 8-week old female C57BL/6 mice were used to establish an LPS-induced ALI model. In brief, mice were anesthetized by isoflurane inhalation, the skin and muscles were incised sequentially to expose the trachea, and 100 μL of 1 mg/kg Lipopolysaccharide (O55:B5, Sigma, USA) was slowly injected into the lungs from the distal end of the trachea using a microinjector. When moist rales appeared in the lungs, indicating that the modeling was successful, the incision was finally sutured. 4 h after infection, 10 μg/mL AdMSC-Exos in 200 μL PBS was administered through the tail vein. The viability and behavior of the mice were observed every 4 h thereafter. 24 h after injury, mice were euthanized by intraperitoneal injection of pentobarbital, and lung tissue and bronchoalveolar lavage fluid (BALF) were taken for analysis. 1 mL of PBS was instilled into the trachea, and BALF was collected by repeated lavage three times. The supernatant was stored after ultra-centrifugation for enzyme-linked immunosorbent assay (ELISA) to measure the concentrations of IL-1β, TNF-α, IL-6 and IL-10. The lower cell pellet was collected and counted, and the alveolar macrophages (anti-CD11c, anti-Siglec F), macrophages (anti-F4/80, anti-CD11b) and neutrophils population (anti-CD11b, anti-Ly6G) were detected by flow cytometry. The lung myeloperoxidase (MPO) levels were determined using mouse MPO ELISA (JCbio, Nanjing, China) following the manufacturer's instructions. For histological analysis, a portion of lung tissue was fixed with 4% paraformaldehyde (PFA), and H&E stained after paraffin embedding. The Leica microscope (Leica, Wetzlar, Germany) was used to randomly select regions (10-15 fields) from 5 μM sections at 200x magnifications. The severity of ALI was assessed by inflammatory cell infiltration and alveolar wall thickening. Another part of the lung tissue was frozen in liquid nitrogen and used for RNA and protein isolation. For the mortality studies, the mice treated with exosome or the controls were injected with a high dose (10 mg/kg, i.t.) of LPS and monitored twice daily for up to 7 days.

To dissect the role of AMs in LPS infection, AMs were depleted from the lungs of C57BL/6 mice via intranasal administration of 100 μL of clodronate liposomes (Yeasen, Shanghai, China) (CL_2_MBP). AMs depletion was confirmed by BAL cell staining. Consistent with previous reports 33, intranasal administration of CL_2_MBP resulted in 90% depletion of AMs compared with PBS-treated control mice.

### Human adipose mesenchymal stem cell isolation and characterization

Human AdMSCs were isolated from healthy donors with approval from the Research Ethics Committee of the Friendship Plastic Surgery Hospital Affiliated of Nanjing Medical University, following informed consent (approval no. 20190501). Adipose tissue was washed extensively with equal volumes of phosphate buffered saline (PBS) and digested with 0.1% collagenase type I (Sigma, USA) at 37 °C for 90 min. The isolated mononuclear cells were cultured in DMEM- (Dulbecco's Modified Eagle Medium-) high glucose (HyClone, USA) supplemented with 10% fetal bovine serum (FBS, Gibco, USA), 100 U/mL penicillin and 100 μg/mL streptomycin (P/S, Gibco, USA) of 1 × 10^6^ cells in a tissue culture dish with a diameter of 10 cm (Corning, USA) at 37 °C with 5% humidified carbon dioxide. The culture medium was exchanged with a fresh medium after 48 h to remove nonadherent cells and cell debris. The medium was changed every 3 or 4 days until the attached cells exhibited the spindle-shaped morphology of MSCs. Upon reaching approximately 90% confluence, the cells were harvested using 0.25% trypsin (Gibco, USA) and were subcultured at a 1:3 or 1:4 ratio for cell proliferation. The passage 3-8 MSCs were used to isolate exosomes.

To analyze surface markers, the cultured AdMSCs were isolated and harvested at a single-cell level. According to the instructions of the manufacturer, corresponding doses of anti-CD34, anti-CD45, anti-CD90, and anti-CD105 monoclonal antibodies (BD Biosciences Pharmingen, San Diego, CA, USA) were added to each tube in turn. Meanwhile, the same type of negative control was established. Then, it was mixed well and incubated at 4 °C for 30 min and detected by flow cytometry (BD Biosciences Pharmingen, San Diego, CA, USA).

To assess whether AdMSCs have the capacity of differentiating into osteoblasts, and adipocytes, the cultured AdMSCs were induced with a specific differentiation media kit (ViVacell Bioscience, Shanghai, China). Briefly, the culture media were replaced with an osteogenic or adipogenic medium when the cells reached 70% confluence. The media were changed every 3-4 days. After 2 weeks, osteogenesis was evaluated by Alizarin Red S calcium staining. Adipogenesis was determined by staining the cells with Oil red O. Images of the stained cells were taken using a Leica microscope (Leica, Wetzlar, Germany).

To knockdown the level of NDUFV2 in AdMSCs, small interfering RNA (Genepharma, Shanghai, China) targeting NDUFV2 was used to infect the cells for 4-6 h. The transfection was completed after 36 h, and the cells were used for subsequent experiments.

### Isolation and characterization of AdMSC-derived exosomes

Exosomes were obtained from supernatants of MSCs cultured 48 h in exo-depleted conditioned medium. To obtain exosomes, the supernatants from MSCs underwent centrifugation at 500 × g for 10 min, 2,000 × g for 20 min, 10,000 × g for 30 min to remove dead cells and cell debris, and then cell-free supernatant were centrifuged at 110,000 × g (Beckman Coulter Optima ultracentrifuge, USA) for 80 min at 4 °C, washed in PBS and subjected to a second ultracentrifugation under similar conditions. The final pellets were resuspended in PBS and stored at -80 °C. The mean size and particle concentration were evaluated using Nanosight NS300 instrument (Malvern Instruments, Worcestershire, UK). Exosomes in PBS were placed on 300-mesh copper grids and stained with 2% uranyl acetate. Images were obtained using a JEM-1010 electron microscope (JEOL, Tokyo, Japan) operated at an acceleration voltage of 100 kV.

### MH-S cell culture

MH-S mouse macrophage cells (ATCC CRL-2019) were maintained in 1640 medium (HyClone, USA) containing 10% FBS, 100 U/mL penicillin and 100 μg/mL streptomycin (Gibco, USA). MH-S cells were pretreated with human adipose-derived mesenchymal stem cell-derived exosomes (10 μg/mL) 30 min in advance, followed by 100 ng/mL LPS (Sigma, USA) stimulation. The effects of stem cell-derived exosomes on mouse alveolar macrophages under LPS stimulation were observed. Cells were collected at different time points for RNA and protein extraction. The expressions of inflammatory factors in the culture supernatant, macrophage typing and mitochondrial function status were further examined.

To knockdown the level of NDUFV2 in MH-S cells, small interfering RNA (Genepharma, Shanghai, China) targeting NDUFV2 was used to infect the cells for 4-6 h. The transfection was completed after 36 h, and the cells were used for subsequent experiments.

### Collection and culture of primary alveolar macrophages

After the mouse alveolar lavage fluid was centrifuged, it was resuspended in DMEM medium (HyClone, USA) containing 10% FBS (Gibco, USA), 100 U/mL penicillin and 100 μg/mL streptomycin (Gibco, USA), and the cell suspension was inoculated into a 6-well plate and incubated at 37 °C with 5% humidified carbon dioxide 2 h.

### Macrophage phenotype analysis by flow cytometry

MH-S cells were immunolabeled with antibodies against surface proteins. The following antibodies were used: anti-MHC Ⅱ allophycocyanin (PE), and anti-CD206 phycoerythrin (APC) (eBioscience, USA). Macrophages were collected and incubated with antibodies in the dark at 4 °C for 30 min and then washed with PBS. The expression of macrophage markers was calculated based on the fluorescence intensity. All of the data were collected by flow cytometry and analyzed using Flow Jo software.

### Macrophage apoptosis and proliferation analysis by flow cytometry

Annexin V staining kit (Sigma, USA) was used to assess apoptosis in MH-S cells. Cells were washed in 1 × binding buffer and stained with FITC-conjugated annexin V and propidium iodide (PI) antibody for 15 min and immediately assessed by flow cytometry. Use CFSE (Thermo, USA) for MH-S cell proliferation assay. The expression of macrophage markers was calculated based on the fluorescence intensity. All of the data were analyzed using Flow Jo software.

### Enzyme-linked immunosorbent assay (ELISA)

The levels of TNF-α, IL-6, IL-1β, and IL-10 in bronchoalveolar lavage fluid of mice and supernatants from MH-S cells were measured using ELISA kits (Lianke, Hangzhou, China). The assay was conducted following the guide of manufacturer's protocols. The absorbance was detected using Epoch microplate reader (BioTek, USA) at 450 nm. The reference length was 570 nm.

### Quantitative Real-Time PCR (qRT-PCR)

Total RNA was extracted using Trizol reagent (Thermo, USA), and 1 μg of total RNA was subjected to reverse transcription of mRNA using HiScript II One Step RT-PCR Kit (Vazyme, Nanjing, China) to generate total cDNA. Quantitative PCR was performed in 96-well plates using the LightCycler 480 SYBR Green I Master mix (Yeasen, Shanghai, China) on a LightCycler 480 System (Roche, USA) under the following conditions: 95 °C for 5 min and 95 °C for 10 s, 45 cycles of 60 °C for 20 s, and 72 °C for 15 s. The sequences of the primers used are listed in Table S1. β-actin was used for normalization. The quantitative expression level was analyzed using the 2^-ΔΔCt^ method.

### Western Blot assay

Total protein was extracted using RIPA buffer (Beyotime, Shanghai, China) containing 1 mM phenylmethylsulfonyl fluoride (PMSF) and protease inhibitor cocktail. Protein concentrations were determined by the BCA protein assay. Total proteins (40 μg) were separated by SDS-PAGE and blotted onto PVDF membranes (Millipore, Massachusetts, USA). After blocking with 5% BSA in TBST, the membranes were incubated with the following primary antibodies at 4 °C overnight; anti-CD9, anti-CD63, anti-TSG101, anti-VDAC, anti-LAMP1 were obtained from Abcam (Abcam, USA); and anti-p38/p-p38, anti-ERK/p-ERK, anti-JNK/p-JNK, anti-p65/p-p65, anti-IKKα/p-IKKα, anti-Iκbα/p-Iκbα, anti-Sirt1, anti-GAPDH, anti-β-actin were obtained from Cell Signaling Technology (CST, USA); and anti-PGC1α, anti-cox15, anti-NDUFV2, anti-ATP5D, anti-ATP5H were obtained from Proteintech Biotechnology (Proteintech, USA); and anti-TFAM was obtained from Absin Bioscience (Absin, Shanghai, China); and anti-TOM20, anti-CALR were obtained from Santa Cruz Biotechnology (Santa Cruz, USA). Thereafter, the membranes were incubated with HRP-conjugated anti-mouse and anti-rabbit IgG (1:4000, CST, USA), respectively, at room temperature for 1 h. ECL reagent was used to develop the membrane and signal was detected on a digital image system (Bio-Rad, USA).

### Mitochondrial staining of adipose-derived mesenchymal stem cells

Adhered AdMSCs were incubated with 2.5 μM Mito Tracker Red fluorescent dye (Thermo, USA) staining solution in DMEM in the dark for 30 min at 37 °C. Following incubation, the cells were rinsed twice with PBS and replaced with fresh medium to continue the culture. After 48 h, the culture medium was collected to extract exosomes.

### Immunofluorescence

The labeled exosomes (10 μg/mL) were incubated with MH-S cells. After 12 h, the cells were fixed with 4% paraformaldehyde, washed with PBS, blocked in 3% BSA for 1 h and reacted with primary antibody against rabbit anti-HSP60 (1:200, Proteintech, USA) overnight. The cover glass was further stained with Alexa Fluor 488 goat anti-rabbit IgG (1:1000, Abcam, USA) for 1 h in the dark. After being washed with PBS, the slices were stained with DAPI (1:1000, Beyotime, Shanghai, China) for 10 min, washed with PBS, and added the anti-fluorescence quencher before mounting onto microscope slides. Finally, cells were observed using Leica TCS SP8 laser scanning confocal microscope (Leica Microsystems Ltd., Wetzlar, Germany). Images were analyzed using LAS X software.

### Immunofluorescence staining after mtDNA deletion

To model severe mitochondrial genome damage, MH-S cells were cultured for 72 h in low-dose ethidium bromide (EtBr) (100 ng/mL) followed by transfer to medium lacking EtBr 34. The exosomes (10 μg/mL) were incubated with mtDNA-deleted MH-S cells. After 12 h, the cells were fixed with 4% paraformaldehyde, washed with PBS, blocked in 3% BSA for 1 h and reacted with 0.5 μM Mito Tracker Red fluorescent dye (Thermo, USA) staining in the dark for 30 min at 37°C. After being washed with PBS, the cell slices were stained with DNA dye Draq5 (1:1000, Abcam, USA) for 5 min, washed with PBS, and added the anti-fluorescence quencher before mounting onto microscope slides. Finally, cells were observed using Leica TCS SP8 laser scanning confocal microscope (Leica Microsystems Ltd., Wetzlar, Germany). Images were analyzed using LAS X software.

### Nucleic acid electrophoresis

All sample DNA was loaded at 30 μL/well and detected via 1% agarose gel electrophoresis at 100 V for 45 min. The DNA ladder used was D0107 (Beyotime, Shanghai, China), and the results were visualized by a digital image system (Bio-Rad, USA).

### *In vivo* imaging of the AdMSC-Exos

A total of 10 μg/mL of MitoRed-Exos resuspended in 100 μL of PBS was intravenously administered. Injection with 100 μL of PBS served as controls. At selected time points after administration, the mice were euthanized, and vital organs were collected and imaged by the IVIS Lumina imaging system (Xenogen Corporation, Hopkinto, MA, USA). Mito Tracker Red: λem = 581 nm, λex = 644 nm.

### Lung tissue immunofluorescence

For immunofluorescence staining, lung tissues were dehydrated in 30% sucrose, embedded with frozen O.C.T., and then cut into 6 μm frozen slices. The frozen slice was blocked in 3% BSA for 1 h and reacted with primary antibody against rabbit anti-F4/80 (1:200, abcam, USA), mouse anti-AQP-5 (1:200, Santa Cruz, USA) and rabbit anti-keratin (1:200, Proteintech, USA) overnight. The slices were further co-stained with Alexa Fluor 488 goat anti-rabbit or goat anti-mouse IgG (1:1000, Abcam, USA) for 1 h in the dark. After being washed, the slices were stained with DAPI (1:1000, Beyotime, Shanghai, China) for 10 min and then imaged through CLSM.

### Degradation of DNA and proteins in exosomes

In order to study the specific components contained in stem cell exosomes, we apply DNase I (Yeasen, Shanghai, China) and Proteinase K (Beyotime, Shanghai, China) to degrade the DNA and protein components of the purified exosomes, and used these protein- and nucleic acid-depleted exosomes to treat MH-S cells. In this experiment, we directly added 1 U/mL DNase Ⅰ or 0.1 mg/mL Proteinase K to the purified exosomes, incubated at room temperature for 30 min, collect the lysate, and centrifuge to take the supernatant for downstream experiments.

### Mito Tracker Green and Red

Mito-Tracker Green is a mitochondrial green fluorescent dye, its ability to locate to mitochondria is not affected by the mitochondrial membrane potential and represents all mitochondria contained in the cell, Mito-Tracker Red is a red fluorescent dye, the accumulation of this dye depends on the membrane potential. It fluoresces when oxidized in the respiratory mitochondria and represent a group of functional mitochondria. Here, we select a group of Green^+^/Red^-^ cells in the flow cytometry results, which means that the mitochondria of the cell function in the gate is impaired. Mouse primary alveolar macrophages and MH-S cells were loaded with 100 nM green-fluorescing Mito Tracker Green (Thermo, USA) for 30 min at 37 °C to measure mitochondrial content, and 0.5 μM Mito Tracker Red (Thermo, USA) for 30 min at 37 °C to detect mitochondrial membrane potential, followed by a wash with warmed staining buffer and detected by flow cytometry. The excitation and emission wavelengths for each fluorescent dye were selected according to the manufacturer's instructions. All data were obtained from experiments with at least three replicates.

### Extracellular flux analysis

Oxygen consumption rates (OCR) and extracellular acidification rates (ECAR) were measured using an XFe96 Extracellular Flux Analyzer (Seahorse Bioscience, USA). Before experiments, MH-S cells were pretreated with exosomes (10 μg/mL) for 0.5 h and then stimulated with LPS (100 ng/mL) for 4 h and analyzed under basal conditions and following treatment with the following agents: the ATP synthase inhibitor oligomycin (1 μM); the mitochondrial oxidative phosphorylation uncoupling agent FCCP (0.5 μM) to uncouple mitochondria; the mitochondrial complex I inhibitor rotenone (100 nM) and the mitochondrial complex III inhibitor antimycin A ( 1 μM). The basal oxygen consumption rate (OCR) was calculated by subtracting the OCR after rotenone and antimycin A treatment from the OCR before oligomycin treatment. The maximal OCR was calculated by subtracting the OCR after rotenone and antimycin A treatment from the OCR measured after addition of FCCP.

### Assessment of mitochondrial DNA copy number by qRT-PCR

Total DNA from MH-S cells was isolated using the FlexiGene DNA kit (Qiagen, Germany). Oligonucleotide probes were designed against 3 different regions of mitochondrial DNA (mtDNA), and 2 regions of genomic DNA. qRT-PCR was performed using the Hieff qPCR SYBR Green Master Mix (Yeasen, Shanghai, China). mtDNA copy number was calculated relative to genomic DNA as previously described 35.

For the analysis of mitochondrial DNA contained in exosomes, DNA from human AdMSC-derived exosomes was isolated with the FlexiGene DNA kit (Qiagen, Germany). After DNA isolation, qRT-PCR was performed according to the manufacturer's protocol (Takara, Japan). Primer sequences are specified in Table s1.

### Extraction of mitochondrial DNA

Mitochondrial were isolated using a Cell Mitochondria Isolation kit (Beyotime, Shanghai, China) according to the manufacturer's instructions. Briefly, cells (2 × 10^7^ cells/mL) in the logarithmic growth phase were harvested and washed twice with ice-cold PBS. Cells were incubated with cell lysis buffer (1 mL cell lysis buffer/2 × 10^7^ cells) for 10 min at 4 °C, before they were homogenized with a glass homogenizer. The cell lysate was centrifuged at 600 × g for 10 min at 4 °C to remove any remaining whole cells, and the supernatant was further centrifuged at 11,000 × g for 10 min at 4 °C. The supernatant was removed and the remaining mitochondrial pellet was resuspended in mitochondrial lysis buffer at 4 °C, and centrifuged at 24,000 × g for 15 min at 4 °C to remove the cell nuclei. The mitochondrial DNA was then extracted using a FlexiGene DNA extraction kit (Qiagen, Germany), and the mtDNA extracts were stored at -80 °C until required.

### ATP measurement

After mouse primary alveolar macrophages and MH-S cells were lysed, the lysate was added to a white 96-well plates (100 μL/well) and treated as required. ATP was assayed using an ATP quantification bioluminescent kit (Beyotime, Shanghai, China) according to the manufacturer's instructions.

### JC‐1 staining

Mitochondrial membrane potential (MMP) was detected with a JC‐1 Staining Kit (Beyotime, Shanghai, China). Cells from different groups were washed with PBS before incubation with a 1 mL mixture of JC‐1 staining fluid in the dark for 30 min. Then, the cells were washed with cold staining buffer. Afterward, the MMP of different samples was detected by fluorescence microscopy (Leica Microsystems Ltd., Wetzlar, Germany).

### FACS analysis of reactive oxygen species (ROS)

MitoSOX red mitochondrial superoxide indicator (Thermo, USA) staining was used to measure the production of reactive oxygen species (ROS) in cell mitochondria. This new type of fluorescent probe can selectively indicate the superoxide anion in the mitochondria of living cells. Before use, add an appropriate amount of DMSO to dissolve the reagent powder according to the MitoSOX probe instructions to prepare a 5 mM staining stock solution, and store at -20 °C in the dark. After pretreatment, mouse primary alveolar macrophage and MH-S cells were trypsinized and stained with 5 μM MitoSOX for 30 min at 37 °C. Wash three times with PBS, collect cells by flow cytometer, and analyze the fluorescence intensity of MitoSOX Red and the percentage of mitochondrial ROS using Flow Jo software. All data were obtained from experiments with at least three replicates.

### Transmission electron microscopy (TEM)

Cells were first fixed with 2.5% glutaraldehyde for 12 h, and post fixed with 1% OsO_4_ for 2 h. The specimen was dehydrated with a graded ethanol series for 20 min at each step, transferred to absolute acetone for 20 min, placed in a mixture of absolute acetone and resin overnight, and embedded in resin at 70 °C for more than 9 h. The specimen was then sectioned with ultratome (LEICA EM UC7, Wetzlar, Germany) and stained with uranyl acetate and alkaline lead citrate for 5-10 min. Electron micrographs were taken on a JEM-1230 (JEOL, Tokyo, Japan) transmission electron microscopy.

### Statistical analysis

All statistical analyses were performed using GraphPad Prism 8.0 and SPSS 22.0. Data were presented as the mean ± SD. A one-way analysis of variance test was used to compare the means of normally distributed parameters (Levene's test with 0.1 as the standard test). When equal variances were assumed, the least significant difference test was selected. Otherwise, Dunnett's T3 test was carried out. A value of *P* < 0.05 was considered significant.

## Results

### Purification, Isolation, and Characterization of Adipose-derived mesenchymal stem cell-derived exosomes

**Figure 1:**
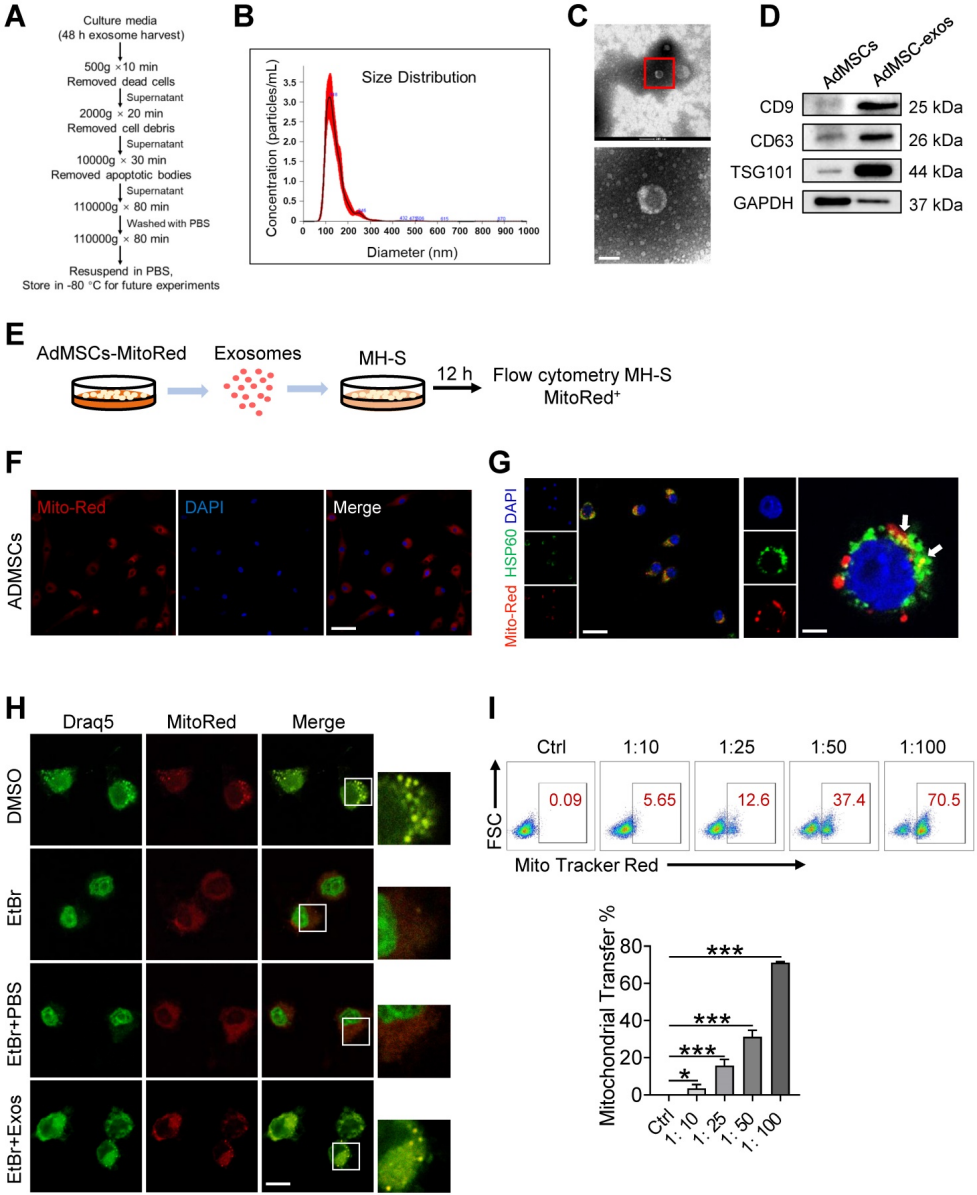
** AdMSC-Exos are capable of transferring mitochondria to macrophages.** (A) To isolate exosomes, conditioned media (CM) were subjected to successive differential centrifugation. (B) Nanoparticle tracking analysis of vesicles derived from AdMSCs. The mean protein concentration and mean particle concentration of the AdMSC-Exos were 2.44 mg/mL and 2.33 × 10^9^ particles/mL, respectively. (C) Transmission electron microscopy analysis of vesicles derived from AdMSCs (Scale bar, 100 nm). (D) Exosome representative markers CD9, CD63 and TSG101 were detected by western blot. Both lanes were loaded with 40 μg of proteins. (E) The experimental schematic of AdMSC-Exos transfers mitochondria to macrophages. Mito Tracker Red was used to pre-label the mitochondria in AdMSCs, and then the exosomes were separated from the cell culture supernatant, and the exosomes were incubated with MH-S cells for 12 h, and the phagocytosis of exosomes by macrophages was analyzed by flow cytometry. (F) The confocal laser scanning microscope micrographs showing Mito-Red staining of mitochondria in adipose mesenchymal stem cells. Scale bar, 50 μm. Mito-red: red, DAPI: blue. (G) The confocal laser scanning microscope micrographs showing that AdMSC-Exos containing stem cell mitochondrial fragments are internalized by macrophages. Left scale bar, 25 μm; Right scale bar, 5 μm. Mito-red: red, HSP60: green, DAPI: blue. (H) The co-localization of mitochondrial DNA and mitochondria in MH-S cells was examined by confocal microscope. Among them, nDNA and mtDNA are dyed green by Draq5, and functional mitochondria are dyed red by Mito Tracker Red. We deleted mtDNA in MH-S cells using low-dose EtBr (Materials and Methods), and then transfer the exosomes to mtDNA-deleted MH-S cells. The EtBr + PBS group is a negative control for exosomes (Scale bar, 10 μm). (I) Representative FACS plots of Mito Red on MH-S cells at increasing amounts of AdMSC-Exos. All the data are expressed as the mean ± SD; *P < 0.05, **P < 0.01, ***P < 0.001.

For the preparation of AdMSC-Exos, we initially isolated AdMSCs from white, subcutaneous adipose tissue that was routinely disposed as surgical waste material 36, 37. Cells were then cultured according to standard stem cell culture and exhibited robust ability to expand and differentiation (Figure S1A-C). After 3-8 generations of passages, exosomes were isolated from culture supernatants by gradient centrifugation (Figure 1A). Transmission electron microscopy (TEM) and nanoparticle tracking analysis (NTA) revealed that exosomes derived from AdMSCs have a diameter range from 50 to 150 nm (Figure 1B), and exhibited distinct biconcave morphological features of exosomes (Figure 1C). Immunoblotting demonstrated that exosome preparations were positive for CD9, CD63 and TSG101, the established exosome markers (Figure 1D).

### AdMSC-Exos transfers mitochondria to macrophages

To explore the possibility that MSCs-derived mitochondrial component could be transferred to recipient macrophages, donor cells (AdMSCs) was firstly labeled with mitochondria probe, Mito Tracker Red. Exosomes were then isolated from the culture supernatant of Mito-labelled AdMSCs, and incubated with murine alveolar macrophage cell line, MH-S cells (Figure 1E). Immunofluorescence imaging revealed that the mitochondrial components in MSCs were labeled by Mito Tracker Red (Figure 1F). Moreover, confocal laser scanning microscopy (CLSM) demonstrated that the labeled mitochondria were internalized and co-localized with mitochondria (label with HSP60) of recipient cells (Figure 1G). To identify the mechanisms involved in AdMSC-derived exosome internalization, we applied CPZ (10 µg/mL) and genistein (200 µM) respectively to inhibit the clathrin and caveolae-mediated endocytosis of exosomes. The result showed that the internalization of AdMSC-Exos was inhibited by treatment of chlorpromazine or genistein (Figure S2), indicated the requirement of endocytosis in this process. To exclude the possibility that the observed mito-Red staining might be due to the dye leakage for staining mDNA in the recipient cells, we then treated MH-S cells with EtBr to deplete mDNA, and incubated them with AdMSC-Exos. As expected, EtBr-treated MH-S cells did not show mtDNA positive signaling unless they received AdMSC-Exos (Figure 1H). The results further supported the transportation of mDNA from AdMSCs to macrophages.

We next evaluated whether the delivery of mDNA was dependent on the dose of exosomes applied. In our system, 2.33 × 10^9^ exosomal particles were obtained from 2 × 10^7^ AdMSCs in 200 mL of cell culture supernatant, corresponding to the protein concentration at 2.44 mg/mL. Accordingly, the protein concentration for 1 × 10^7^ exosomal particles was about 10 μg/mL. In Figure 1I, we incubated MH-S: exosomes at the ratio of 1:10, 1:25, 1:50, and 1:100 respectively, which led to 3.5, 15.6, 31.2, and 71.1% mito-red positive staining in the recipient cells, supporting the dose-dependent transportation of MSC-derived mitochondrial components to macrophages. Taken together, these findings indicated that AdMSC-derived exosomes can transfer mitochondrial ingredients into alveolar macrophages, which were then co-localize with mitochondria in the recipient cells.

### Human mitochondria DNA transfers through AdMSC-exos and improves macrophage mitochondrial fitness

**Figure 2:**
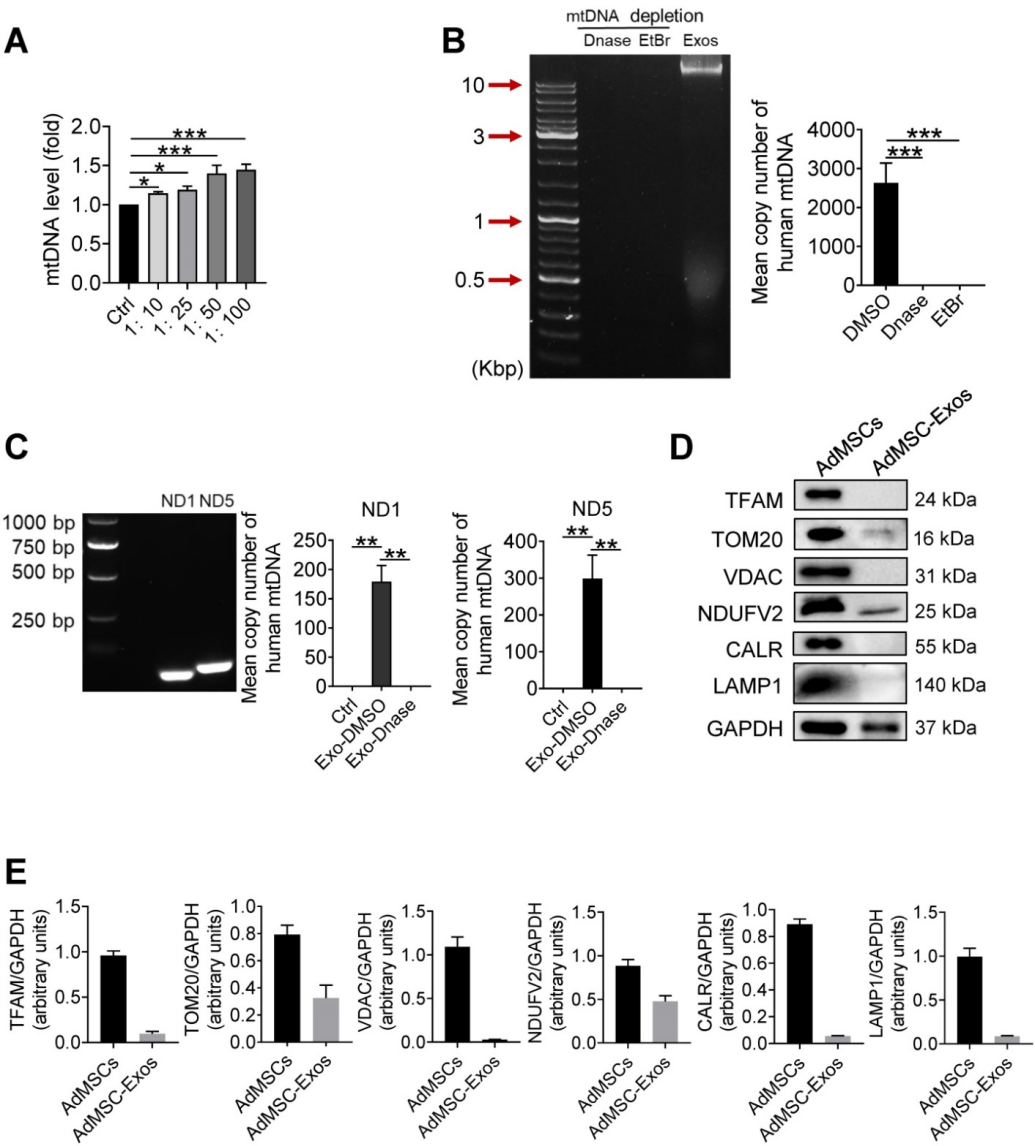
** Human mitochondria DNA transfers through AdMSC-exos and improves macrophage mitochondrial fitness.** (A) Mitochondrial DNA expression on MH-S cells at increasing amounts of AdMSC-Exos. (B) Left panel, representative agarose gel electrophoresis image of the 16.5-kbp whole mtDNA genome from purified AdMSC-Exos. Right panel, the average number of copies of human mitochondrial DNA in exosomes derived from human adipose-derived mesenchymal stem cells. After treatment with Deoxyribonuclease (DNase) and ethidium bromide (EtBr), mtDNA in AdMSC-Exos is deleted. (C) Left panel, the expression of human mtDNA PCR amplified product 109-bp ND1 and 154-bp ND5 detected in human MSCs-derived exosomes. Right panel, after transferring AdMSC-Exos ± DNase to MH-S cells, qPCR detects the copy number of human mitochondrial DNA in MH-S cells. (D) AdMSC and purified AdMSC-Exos were blotted for proteins associated with mtDNA (TFAM), for proteins located in the outer mitochondrial membrane (TOM20 and VDAC), the inner mitochondrial membrane (NDUFV2), the endoplasmic reticulum (CALR) and the lysosomal (LAMP1). (E) The histogram shows the relative ratio of the expression level of the target protein to that of an internal reference. All the data are expressed as the mean ± SD; *P < 0.05, **P < 0.01, ***P < 0.001.

In order to further clarify which mitochondrial components (especially mtDNA) from AdMSC-Exos mediated immune regulatory and lung protective role, we next detected the changes of mitochondrial DNA in MH-S cells after Exo-transferring. As shown in Figure 2A, the mitochondrial DNA level of MH-S cells was elevated upon receiving exosomes. To determine whether it is the carryover of mtDNA from AdMSC-Exos or the mitochondria increase in recipient cells, we detected the expression of human-derived mtDNA in AdMSC-Exos. Agarose gel electrophoresis demonstrated human mtDNA (16.5 kb) in purified EVs, which was largely abrogated upon the treatment of DNase. Additionally, the exosomes released from mtDNA-depleted AdMSCs by treating with a lower level of EtBr (100 ng/mL) showed unnoticeable level of mtDNA (Figure 2B), substantiating the harboring of human mtDNA in AdMSC-Exos. Using human-specific mtDNA primers, we showed PCR amplification of mitochondrial genes (ND1, ND5) in AdMSC-Exos, which was abrogated upon DNase digestion. Accordingly, qPCR analysis indicated that human mDNA (ND1, ND5) were specifically amplified in MH-S cells after incubation with AdMSC-Exos but not DNase-treated exosomes (Figure 2C).

It is well known that mtDNA is a damage-associated molecular pattern whose recognition receptors in macrophages are AIM or cGAS (TLR9 on cell membrane) receptors 38, 39. We next observed differences between exosomal-mtDNA and free-mtDNA in activating sterile inflammation in macrophages. The results showed that purified free-mtDNA did not reduce LPS-induced inflammatory response compared with ADMSC-exo-mtDNA (Figure S3). We speculate that this may be due to cells actively preventing the packaging of pro-inflammatory, oxidized mitochondrial material into exosomes, or that MSCs inhibit Toll-like receptor signaling and macrophage activation by shedding microRNA-containing exosomes. Since mtDNA generally co-exist with associated proteins, such as mtDNA-binding protein and other related proteins to maintain stability or facilitate replication, we also detected related proteins in purified exosomes. As shown in Figure 2D and 2E, AdMSC-Exos exhibited mitochondrial outer membrane protein TOM20 and mitochondrial inner membrane protein NDUFV2, but without lysosomal and endoplasmic reticulum marker proteins. Thus, it was likely that the harboring of mtDNA-binding and packaging protein TOM20 and NDUFV2 might bolster the mtDNA structure in AdMSC-Exos, which may be one of the reasons why ADMSC-exo-mtDNA allows macrophages to maintain their immune tolerance (attenuate their inflammatory response). Taken together, our data indicated that AdMSC-Exos restored the mtDNA levels in recipient macrophages, and this effect was partially due to the transfer of the mtDNA.

### Transferring of mitochondrial component through AdMSC-Exos improves macrophage mitochondrial function

**Figure 3:**
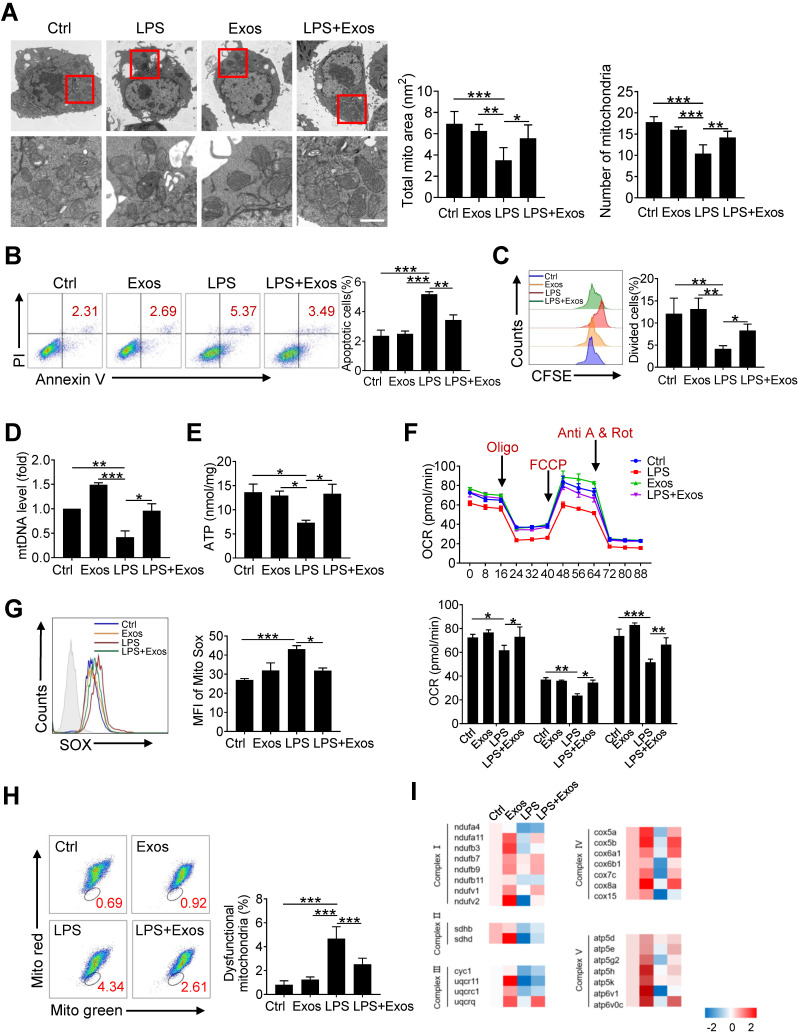
** Transferring of mitochondrial component through AdMSC-Exos improves macrophage mitochondrial function.** (A) Representative transmission electron micrographs (TEM) showing mitochondrial amount, morphology and cristae. The histogram represents the quantification of the size and number of mitochondria. Scale bar, 1 μm. (B) Flow cytometry analysis of the effect of exosomes on MH-S cell apoptosis. (C) Flow cytometry analysis of the effect of exosomes on the proliferation of MH-S cells. (D) Mitochondrial DNA expression in MH-S cells. (E) Expression of mitochondrial ATP production in MH-S cells. (F) Effects of AdMSC-Exos on oxidative phosphorylation of MH-S cells detected by Extracellular flux analysis. (G) Flow cytometry and quantification of mitochondrial reactive oxygen species (ROS) levels by staining with Mito Sox. (H) Flow cytometry of mitochondria staining with Mito Tracker Red and Mito Tracker green (Green^+^/Red^-^). (I) Heatmap showing the expression of mitochondrial respiratory chain complex-related genes in MH-S cells detected by qPCR. Primer sequences are reported in Table s1. All the data are expressed as the mean ± SD; *P < 0.05, **P < 0.01, ***P < 0.001.

Since it has been demonstrated that morphology and function of mitochondria in alveolar macrophages was affected during ALI onset, we then wondered whether AdMSCs served as the donors of mtDNA to relieve mitochondrial stress in macrophages in this setting. For this, MH-S cells were firstly subjected to LPS stimulation and their mitochondrial morphology and function were examined. As expected, transmission electron microscopy revealed that the number of mitochondria in macrophages was decreased following endotoxin exposure, with mitochondrial cristae disorganized and vacuoles appearing in some mitochondrial cavities (Figure 3A). By contrast, macrophages that were pre-treated with AdMSC-Exos displayed mitochondria with increased amounts and improved morphology as well as increased mitochondrial membrane potential (Figure S4). Moreover, AdMSC-Exos administration decreased the apoptotic (AnV^+^/PI^-^) rate and improved the proliferative capability of MH-S cells (Figure 3B, C). Also, mtDNA content and ATP generation that were compromised by LPS stimulation were remarkably increased upon the application of AdMSC-Exos (Figure 3D, E). The data thus indicated that the delivery of mtDNA via exosomes increased mtDNA level and energy-generating capability of macrophages that were compromised following endotoxin exposure.

To study the effect of AdMSC-Exos on macrophage bioenergetics, we also analyzed oxygen consumption rates (OCR), indicative of mitochondrial function, through Seahorse XF Analyzers. In line with the disturbed mitochondria induced by LPS, the level of OCR was down-regulated in LPS-stimulated MH-S cells, which was alleviated upon the pre-treatment of AdMSC-Exos (Figure 3F).In addition, AdMSC-Exos application lessened mitochondrial ROS stress and decreased the percent of macrophages with dysfunctional mitochondria, as detected by MitoRed/Green Tracker staining (Figure 3G, H). Associated with this, the expression of a network of mitochondrial genes, representative of respiratory chain complexes Ⅰ, Ⅱ, Ⅲ, Ⅳ and Ⅴ, was elevated in MH-S cells upon the treatment of AdMSC-Exos (Figure 3I).

Congruent with the report about the impaired expression of NDUFV2 during LPS-induced lung injury 40, we noted that the expression of NADH: ubiquinone oxidoreductase core subunit V2 (NDUFV2) was reduced upon LPS stimulation (Figure S5A). The transfer of mitochondria via AdMSC-Exos caused a resumption of NDUFV2, leading to the mitigation of mitochondrial pathology in LPS-challenged macrophages. To further confirm the mitochondria-resuming function of exosomes, we then knocked down NDUFV2 in MH-S cell incubated them with AdMSC-Exos. Expectedly, the transfer of AdMSC-Exos efficiently increased the expression of NDUFV2 (Figure S5B, C), accompanied by reduced expression of pro-inflammatory cytokines in NDUFV2-silenced MH-S cells following LPS stimulation (Figure S5D). In parallel, mitochondria-protecting property of AdMSC-Exos, as evidenced by elevated mtDNA, ATP generation, alleviated mROS stress and dysfunctional mitochondria, was partially resumed in these cells (Figure S5E-H). Thus, it appeared that AdMSC-Exos replenished damaged mitochondrial genes such as NDUFV2 in LPS-challenged macrophages, and thereby improved mitochondrial function and blunted inflammatory response.

Next, we knocked down NDUFV2 in AdMSCs (Figure S6A). Exosomes from control (NC) or NDUFV2-silenced AdMSCs were then collected to treat MH-S cells. The results showed that as compared with that from NC-treated AdMSCs, the ability of exosomes from NDUFV2-silenced AdMSCs to inhibit the expression of LPS-induced inflammatory cytokines was attenuated (Figure S6B). Accompanied with this, exosomes from NDUFV2-silenced AdMSCs failed to restore mitochondrial function in macrophages, as detected by MitoRed/Green staining and ATP generation (Figure S6C-F). The results thus further supported that transfer of mitochondrial component from AdMSCs was critical for the protective effect of AdMSC-Exos.

### AdMSC-Exos promotes functional shift of endotoxin-challenged macrophages to anti-inflammatory phenotype via mitochondria transfer

**Figure 4:**
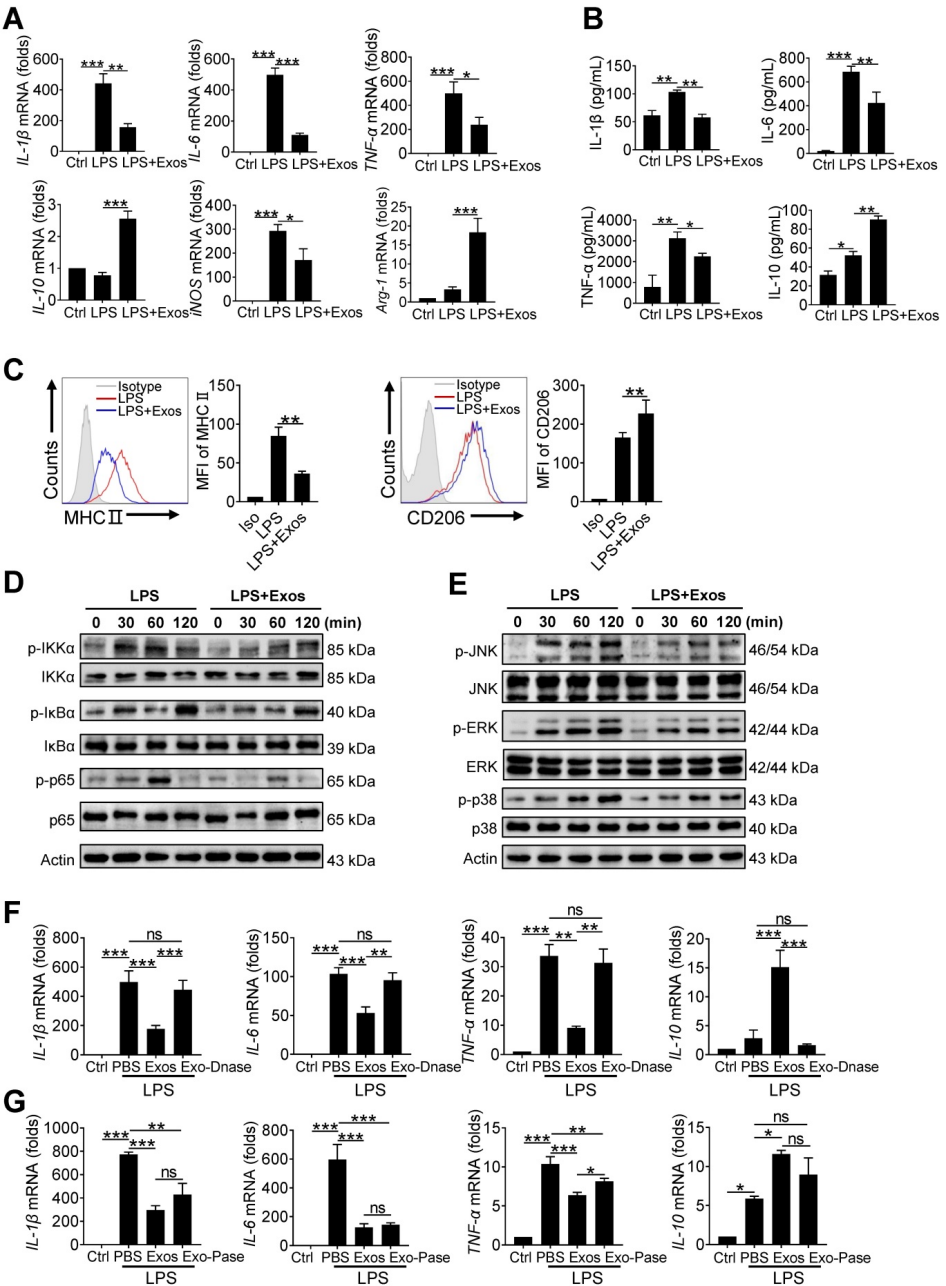
** AdMSC-Exos promotes functional shift of endotoxin-challenged macrophages to anti-inflammatory phenotype via mitochondria transfer.** MH-S cells were pretreated with AdMSC-Exos or PBS for 30 min, and then subjected to LPS (100 ng/mL) stimulation for the time periods, as indicated. (A) Relative mRNA of pro-inflammatory and anti-inflammatory cytokines were assayed by qPCR. (B) Expression of MHC II and CD206 on MH-S cells 12 h post-LPS exposure was detected by flow cytometry. Representative histograms and average relative mean fluorescence intensity (MFI) are depicted. (C) Relative levels of pro-inflammatory and anti-inflammatory cytokines were assayed by ELISA. (D, E) Western blot analysis of phosphorylated and total IKKα, IκBα, p65, JNK, ERK and p38 levels in MH-S cells that were pretreated with AdMSC-Exos or PBS for 30 min and then stimulated with LPS for the indicated time periods. (F) The effect of nucleosome inhibition on the anti-inflammatory effect of AdMSC-exos. (G) The effect of proteasome inhibition on the anti-inflammatory effect of AdMSC-exos. All the data are expressed as the mean ± SD; *P < 0.05, **P < 0.01, ***P < 0.001.

Mitochondria exert a wide range of physiological functions in addition to energy production. Particularly, they are essential in metabolically programing immune cells and remolding cellular phenotype and function 41-43. We next assessed whether AdMSC-exo-mediated mitochondria transfer would induce the phenotypic shift in macrophages in response to LPS stimulation. The results showed that LPS-treatment induced highly expression of proinflammatory cytokines, including IL-6, TNF-α, and IL-1β, as well as key M1 marker gene iNOS, which however was profoundly reduced by AdMSC-Exos treatment. On the contrary, the expression of M2-featured genes such as IL-10 and Arg-1 that was suppressed by LPS stimulation was markedly increased in macrophages following AdMSC-Exos administration (Figure 4A, B). In parallel, flow cytometry analysis revealed that AdMSC-Exos down-regulated MHC II (M1 marker) level, but enhanced the expression of CD206 (M2 marker) in LPS-stimulated MH-S cells (Figure 4C). Together, AdMSC-Exos rendered LPS-stimulated macrophages shifting from M1 proinflammatory to M2-polarized anti-inflammatory phenotype.

It has been reported that ROS generated after mitochondrial oxidative stress in macrophages can promote the activation of inflammatory signaling pathways such as NF-κB, and these inflammatory macrophages can aggravate tissue damage 44, 45. To understand the mechanism underlying this functional transformation, we next analyzed the signaling pathways involved, such as nuclear factor kappa-B (NF-κB) and mitogen-activated protein kinase (MAPKs). As shown in Figure 4D, inflammatory pathways were activated after LPS stimulation, and cellular stress was consistent with mitochondrial damage. AdMSC-Exos treatment impeded the activation of p65/NF-κB and its upstream signaling molecules in macrophages upon LPS stimulation. Also, LPS-stimulated phosphorylation of JNK, ERK, and p38 kinase was suppressed following AdMSC-Exos application (Figure 4E). We thus concluded that AdMSC-Exos treatment blunted the key signaling pathways required for expression of pro-inflammatory mediators, leading to the reverse of proinflammatory phenotype of macrophages induced by LPS stimulation.

At the same time, we also found that the DNase-treated AdMSC-Exos demonstrated to largely abrogate its inflammation-inhibiting effect when applied to MH-S cells (Figure 4F). However, the AdMSC-Exos treated with protease still maintained the anti-inflammatory effect (Figure 4G), implying that protein components were not essential for immunoregulatory function of the exosomes.

### The delivery of AdMSC-Exos alleviates lung inflammation and injury in mice

**Figure 5:**
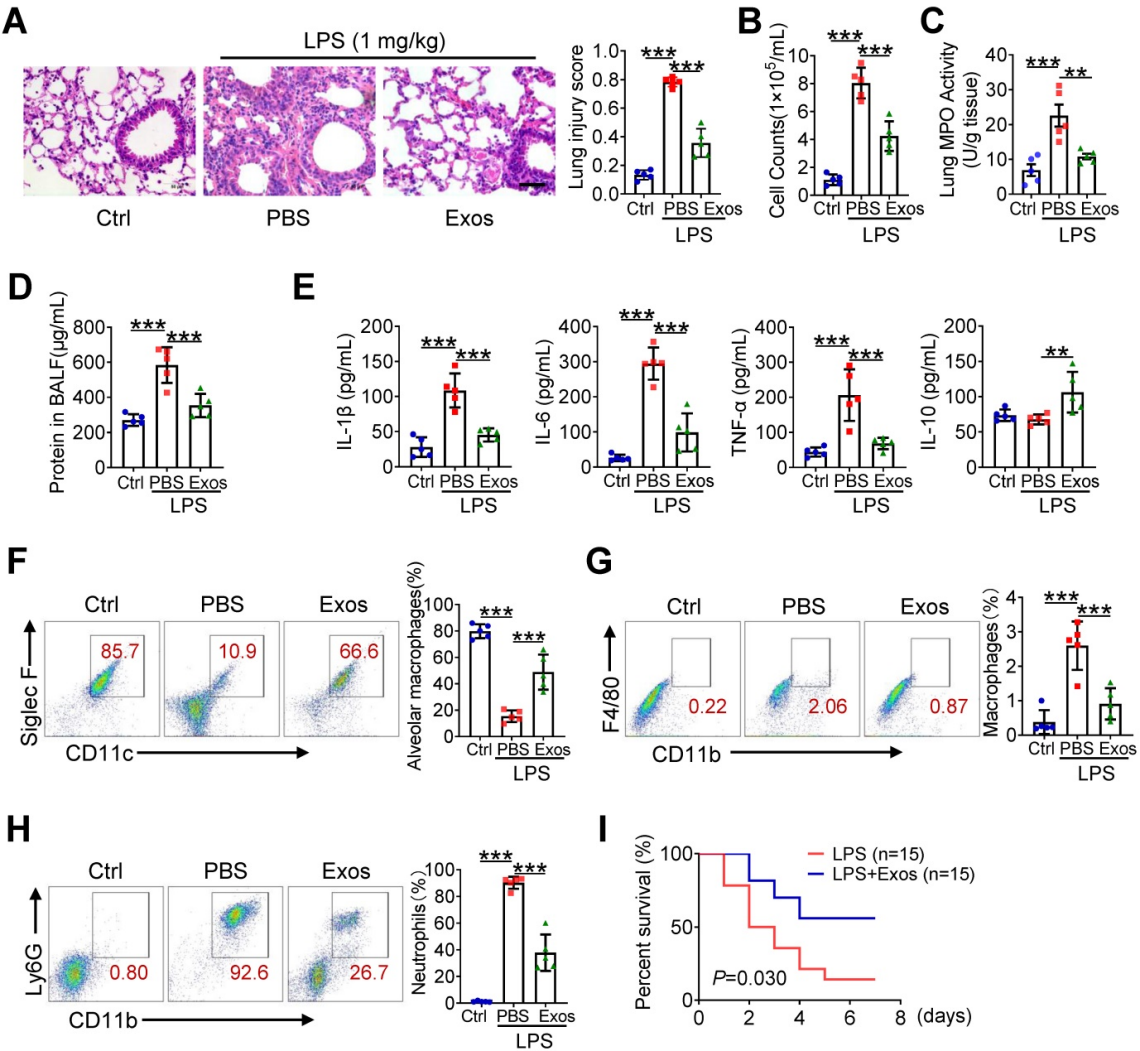
** The delivery of AdMSC-Exos alleviates lung inflammation and injury in mice.** C57BL/6 mice (n = 5 mice/group) were challenged with LPS (1 mg/kg, intratracheally) for 4 h, and then tail vein injected with PBS or Exos (10 μg/mL). 24 h later, mice were sacrificed and subjected to the functional analysis. (A) Representative H&E staining of lung tissues (Scale bar, 50 μm). The histogram shows the lung tissue pathological damage score. (B) BALF cell counts; (C) MPO level in lungs; (D) total protein level; and (E) cytokine concentration in the BAL fluid. (F) Flow cytometry analysis of Alveolar macrophages (CD11c^+^ Siglec F^+^) in BAL fluid (Gate on CD11b^-^ CD64^+^). (G) Flow cytometry analysis of macrophages (CD11b^+^ F4/80^+^) in BAL fluid. (H) Flow cytometry analysis of neutrophils (CD11b^+^ Ly6G^+^) in BAL fluid. (I) Survival rate of the mice that were challenged with LPS at a high dose (10 mg/kg, i.t.) and then treated with Exos or PBS (Injection via tail vein). Kaplan-Meier survival plots were depicted (n = 15 mice/group). All the data are expressed as the mean ± SD; *P < 0.05, **P < 0.01, ***P < 0.001.

Next, we assessed the protective role of AdMSC-Exos in mice model of LPS-induced ALI. For this, C57BL/6 mice were intratracheally instilled with 1 mg/kg LPS, followed by administration of AdMSC-Exos or PBS via tail vein injection (Figure S7A). Compared with vehicle-treated endotoxic mice, the animals receiving AdMSC-Exos exhibited significantly alleviated lung damage, as evidenced by reduced inflammatory cell infiltration and alveolar wall thinning (Figure 5A). Additionally, we found that small amounts of exosomes were internalized into ACE-I and ACE-II cell by CLSM (Figure S7B), suggested that exosomes repaired alveolar epithelial cells to a certain extent to help lung tissue remodeling. Consistently, cell counts and protein leakage in BALF, as well as MPO activity in lung tissues, were remarkably decreased in mice treated with AdMSC-Exos (Figure 5B-D).The production of M1 type cytokines including IL-1β, IL-6 and TNF-α was reduced, whereas the level of M2 signature factor IL-10 was increased upon AdMSC-Exos treatment relative to vehicle injection (Figure 5E, S7C). Furthermore, flow cytometry showed that application of AdMSC-Exos increased the percent of alveolar macrophages (AMs), the subset essential for tissue homeostasis and repair during ALI. Conversely, the amounts of proinflammatory neutrophils and monocyte-derived macrophages were decreased upon AdMSC-Exos treatment (Figure 5F-H). The data thus revealed the anti-inflammatory and tissue protective role for AdMSC-Exos during severe lung inflammatory disorders. To extend this finding, we also assessed the protection of AdMSC-Exos in a murine model of endotoxic shock. As shown in Figure 5I, the application of AdMSC-Exos increased the survival rate of mice following injection of a lethal dose of endotoxin (10 mg/kg, i.t.).

We additionally constructed DNA-deplete exosomes to assess their *in vivo* function using mice model of ALI. As a result, DNA-depleted exosomes were shown not as efficient as vehicle-treated ones in reducing inflammatory response and alleviating lung injury (Figure S8). Together, our data indicated that AdMSC-Exos alleviated lung inflammation, improved tissue integrity, and conferred a profound protection for mice against ALI.

### AdMSC-Exos treatment improves macrophage mitochondrial function *in vivo*

**Figure 6:**
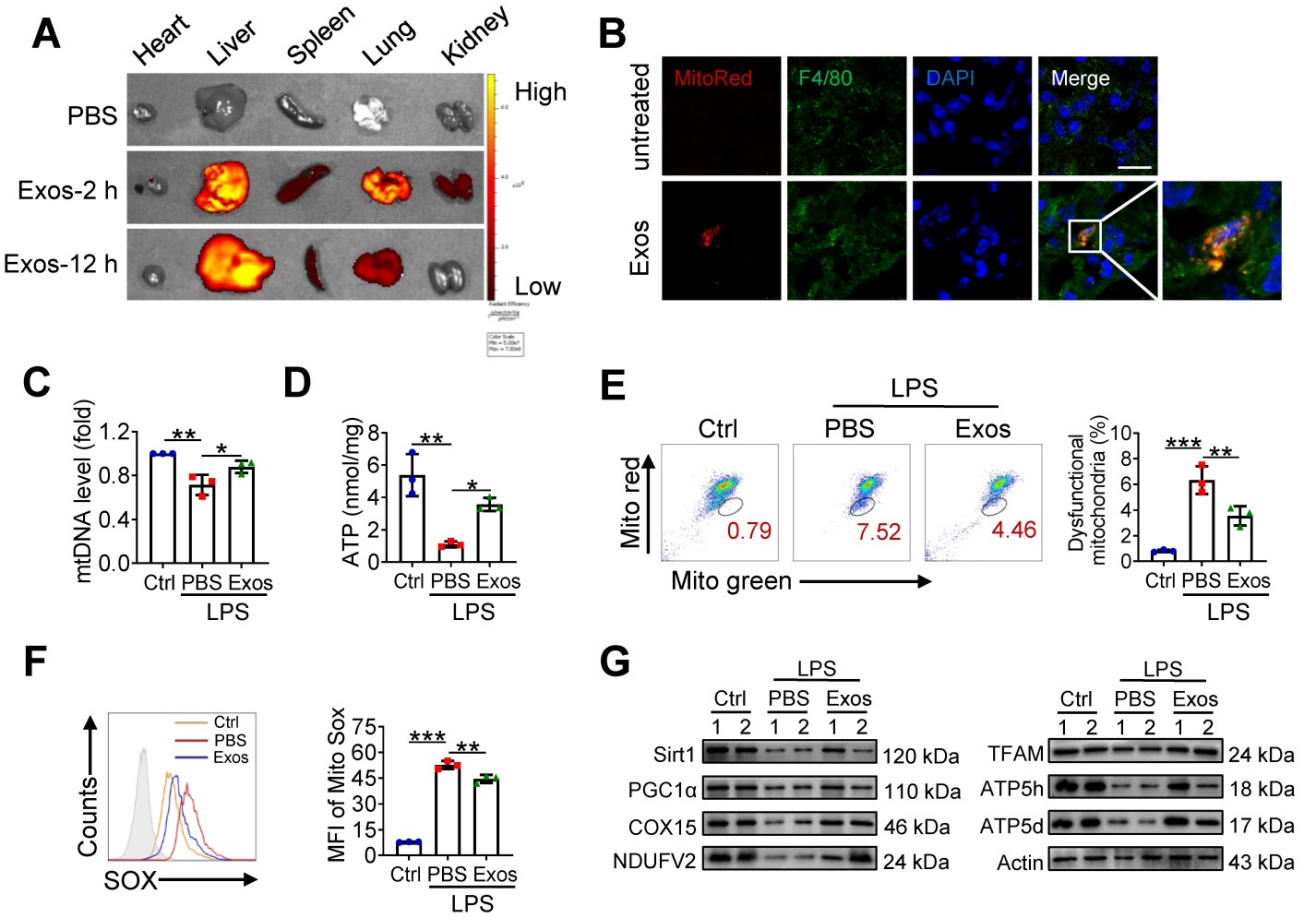
** AdMSC-Exos treatment improves macrophage mitochondrial function *in vivo.*** (A) *Ex vivo* images of vital organs at 2 and 12 h after MitoRed-exos post injection. (B) Representative confocal laser scanning microscopy micrographs showing the colocalization of F4/80 immunostaining (green) with internalized AdMSC-Exos (red). Scale bar, 10 μm. DAPI: blue. (C) Mitochondrial DNA expression in Mouse alveolar macrophages. (D) Assay of ATP generation in Mouse alveolar macrophages. (E) Effects of AdMSC-Exos on mitochondrial viability and quantity in Mouse alveolar macrophages by flow cytometry. (F) Flow cytometry and quantification of Mouse alveolar macrophages mitochondrial ROS levels by staining with Mito Sox. (G) Expression of mitochondrial-associated protein in mouse alveolar macrophages. Shown are representative data from two independent experiments. All the data are expressed as the mean ± SD; *P < 0.05, **P < 0.01, ***P < 0.001.

Given the above findings that AdMSC-Exos treatment partially corrected mitochondrial abnormity of macrophages challenged by LPS, we then explored whether the protective effect of AdMSC-Exos against ALI was related to its regulation of alveolar macrophages. We initially detected MitoRed-labeled AdMSC-Exos in macrophages of lungs, implying that AdMSC-Exos was taken by pulmonary macrophages (Figure 6A-B). As a result, the integrity and function of mitochondria disrupted by endotoxin challenge were improved in alveolar macrophages from mice receiving AdMSC-Exos treatment as demonstrated by increased copy of mtDNA and ATP generation, as well as decreased ratio of dysfunctional mitochondria and lessened ROS release (Figure 6C-F). Furthermore, the levels of mitochondria key molecules including those related with mitochondrial biosynthesis and homeostasis (PGC-1α, TFAM and Sirt1), as well as those associated with mitochondrial respiratory chain (COX15, NDUFV2, ATP5d and ATP5h) were largely resumed upon AdMSC-Exos treatment in alveolar macrophages (Figure 6G). The data thus demonstrated that AdMSC-Exos treatment improved mitochondrial function and immune homeostasis of pulmonary macrophages in LPS-challenge mice.

### The therapeutic effect of AdMSC-Exos on ALI largely depends on alveolar macrophages

**Figure 7:**
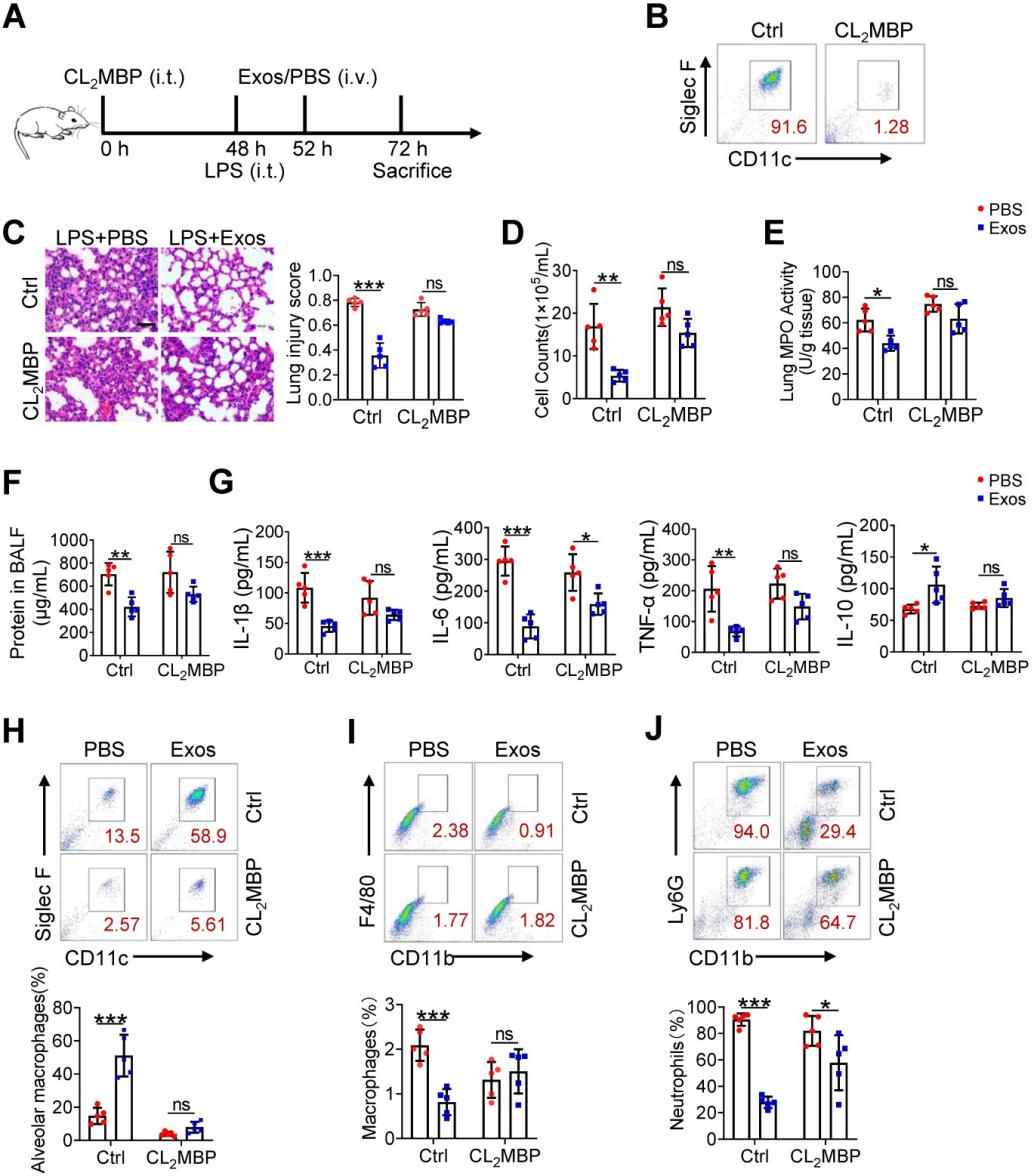
** The therapeutic effect of AdMSC-Exos on ALI largely depends on alveolar macrophages.** (A) The experimental schematic of Alveolar macrophage depletion experiment (n = 5 mice/group). (B) Flow cytometry analysis of Alveolar macrophages (CD11c^+^ Siglec F^+^) in the BAL fluid from different groups. (C) Representative H&E staining of lung tissues (Scale bar, 50 μm). The histogram shows the lung tissue pathological damage score. (D) BALF cell counts; (E) MPO level in lungs; (F) total protein level in the BAL fluid. (G) cytokine concentration in the BAL fluid. (H) Flow cytometry analysis of Alveolar macrophages (CD11c^+^ Siglec F^+^) in BAL fluid (Gate on CD11b^-^ CD64^+^). (I) Flow cytometry analysis of macrophages (CD11b^+^ F4/80^+^) in BAL fluid. (J) Flow cytometry analysis of neutrophils (CD11b^+^ Ly6G^+^) in BAL fluid. All the data are expressed as the mean ± SD; *P < 0.05, **P < 0.01, ***P < 0.001.

Although alveolar macrophages have been appreciated as major player in eradication of deletions agent and maintaining lung homeostasis, their importance in AdMSC-exo-mediated protection of ALI needed to be further defined. For this, we depleted alveolar macrophages in endotoxin-challenged mice by intratracheal administration of clodronate liposomes (Figure 7A). The result showed that a single instillation of clodronate but not control liposomes effectively eliminated macrophages in murine lungs (Figure 7B). Consequently, the anti-inflammatory and tissue-protecting effects of AdMSC-Exos were largely abolished in macrophage-depleted mice, as evidence by elevated BALF cells counts and protein concentrations, increased lung MPO activity, exacerbated lung histopathology, and the reversed expression of M1/M2-related markers (Figure 7C-G). Flow cytometry consistently showed that after depletion of lung macrophages, AdMSC-Exos failed to reduce lung infiltration of proinflammatory neutrophils and mononuclear macrophages in LPS-challenged mice (Figure 7H-J). Interestingly, we noted that the percentage of BALF neutrophils upon CL_2_MBP treatment was not elevated to the control level, which correlated non-completely-rescued IL-6 expression upon macrophages depletion (Figure 7G). The reason for that might be macrophage-unrelated cells contributing to neutrophils induction and IL-6 expression. Alternatively, elimination of AMs impaired the clearance of apoptotic neutrophils from the inflamed lungs, which may affect neutrophil presence and hence IL-6 expression 46, 47. Taken together, these results suggested that alveolar macrophages appeared to be the major effectors responsible for the protection of AdMSC-Exos against ALI.

## Discussion

Acute lung injury (ALI) is generally accompanied by extensive airway inflammation, hypoxemia, tissue disorganization, and lack of effective treatment 48. LPS, the essential cell wall component of Gram-negative, is among the most potent causes 49. In this study, we observed that 24 h after LPS instillation, the mice exhibited the increase in respiratory rate and body temperature, loss in appetite and body weight, and retarded movement, indicating the physiological function of mice were severely affected by intratracheal endotoxin administration. The inflicted mice displayed hallmarks of lung inflammation and injury, as demonstrated by collapsed alveolar structure, thickened alveolar septum, altered membrane transparency, and infiltration of a large number of inflammatory cells. The critical condition is reminiscence of clinical manifestation associated with severe airway infection diseases such as COVID-19, exaggerating the pressing need to invent new potential therapeutics for ALI and related pathology.

Mesenchymal stem cells (MSCs) are multipotent progenitor cells derived from a variety of tissues including adipose tissue, bone marrow, umbilical cord, placenta and amniotic fluid 50. Adipose-derived mesenchymal stem cells (AdMSCs) possess multiple advantages including ethical access, abundant source, and low immunogenicity and renewal properties 51. Therefore, AdMSCs has been experimentally tested to repair damaged tissues and treat some critical disorders, such as degenerative neurologic diseases, liver and kidney injury, and diabetes 52-55. However, to date only a few clinical trials with MSCs are approved because there still exist some unresolved issues about doses, purity and safety of MSCs when applied.

Exosomes from MSCs have recently emerged as a new cell-free alternative for treating inflammatory diseases 56, 57. Compared with stem cells, AdMSC-Exos exhibit low immunogenicity, lack of tumorigenicity, high clinical safety and minimal ethical concerns. Generally sized between 40-150 nm, AdMSC-Exos contain a large quantity of cellular intergradient such as proteins, nucleic acids, and lipids, which are presumably derived from the donate stem cells 58. In this study, we demonstrated that AdMSC-Exos exhibited the immune regulatory and lung protective effects in endotoxin-challenged mice. The effects were achieved through the transferring of mitochondrial components (mtDNA specially) from MSCs to stressed macrophages in lungs. In past publications free mtDNA is known to act as a DAMP to stimulate TLR9, cGAS-STING or AIM2-related pathways to induce a pro-inflammatory state or apoptosis 38, 39. Our results also demonstrated that cell-free-mtDNA has no contribute to cellular inflammatory responses, but mtDNA-containing exosomes show a strong protective effect. In this regard, studies have demonstrated that cells would actively prevent the packaging of pro-inflammatory, oxidized mitochondrial material into exosome that might act as damage-associated molecular patterns (DAMPs). Pathogenic conditions however might through OPA1 and Snx9 disturb this selective packing and release mitochondrial DAMP 59. Alternatively, MSCs were shown to suppress Toll-like receptor signaling and macrophage activation via shedding microRNA-containing exosomes 60. However, currently our understanding about the selective packing of mitochondrial components in exosomes is preliminary. It is likely that stressed or inflammatory conditions of donor cells, or the impairment of cargo-sorting machinery may generate pro-inflammatory mtDNA, or DAMPs.

Mitochondrial OXPHOS not only provides bioenergetics and bio-intermediates for cellular growth, but also determines cellular identity through metabolic-epigenetic modulation 61-63. Resumption of mitochondrial integrity and biogenesis is therefore critical for maintaining normal condition of macrophages and preventing over-reaction to LPS stimulation. In current study, we demonstrated that upon applying recipient cells such as alveolar macrophages, AdMSC-Exos can be directly fused to cytoplasmic membrane of the recipient cell, facilitating their intracellular internalization through endocytosis or phagocytosis. AdMSC-Exos elevated the level of mtDNA, increased mitochondrial membrane potential, OXPHOS activity and ATP generation, while reliving mROS stress in LPS-challenged macrophages. Consistent with the fact that disturbed mitochondrial activity was associated with proinflammatory M1 macrophages, restoring mitochondrial integrity via AdMSC-Exos promoted macrophages shift to anti-inflammatory phenotype. Accordingly, LPS-stimulated release of IL-1β, TNF-α and iNOS was reduced while the production of anti-inflammatory cytokines IL-10 and Arg-1 was resumed upon AdMSC-Exos treatment. We thus link the mitochondrial repairing with immune homeostasis, which likely constitutes the central mechanism of protective effect of AdMSC-Exos. Notably, alveolar macrophages play an indispensable role in this regulatory process, as the protective role for AdMSC-Exos was largely abrogated when alveolar macrophages were depleted. We thus not only identify alveolar macrophages as the essential target by AdMSC-Exos, but also suggest a stem cell-based strategy that might be exploited to treat inflammatory lung diseases.

In summary, our data demonstrate that exosomes from human AdMSCs, primarily through transferring of mitochondrial DNA, promote macrophages metabolic and immune homeostasis and alleviate the severity of ALI. The use of exosome biologics for acute lung injury is of timely importance due to pressing requirements for effective treatment of critical lung diseases such as COVID-19.

## Supplementary Material

Supplementary figures and tables.

## Abbreviations

AdMSCs: adipose-derived mesenchymal stem cells
ALI: acute lung injury
AMs: alveolar macrophages
ARDS: acute respiratory distress syndrome
ATP: adenosine-triphosphate
BALF: bronchoalveolar lavage fluid
CL_2_MBP: dichloromethylene-bisphosphonate
CLSM: confocal laser scanning microscopy
DAPI: 4',6-diamidino-2-phenylindole
ECAR: extracellular acidification rate
EtBr: ethidium bromide
Exo: exosome
IL: interleukin
LPS: lipopolysaccharide
MAPK: Mitogen-activated protein kinase
MMP: mitochondrial membrane potential
MPO: myeloperoxidase
mtDNA: Mitochondrial DNA
M1: M1 type macrophages
M2: M2 type macrophages
NDUFV2: NADH dehydrogenase (ubiquinone) flavoprotein 2
nDNA: Nuclear DNA
NF-κB: nuclear factor kappa-B
NTA: nanoparticle tracking analysis
OCR: oxygen consumption rate
PBS: phosphate-buffered saline
ROS: Reactive oxygen species
siRNA: small interfering RNA
TEM: transmission electron microscopy
TNF-α: tumor necrosis factor alpha
