# Supplementary material for: AdMSC-derived exosomes alleviate acute lung injury via transferring mitochondrial component to improve homeostasis of alveolar macrophages
Source: Theranostics. 2022 Mar 21;12(6):2928–47. doi: 10.7150/thno.69533 (PMC8965475; doi:10.7150/thno.69533)
Supplement: Supplementary file 1 — Supplementary figures and tables. [file thnov12p2928s1.pdf]

FIGURE S1. Isolation and characterization of AdMSCs

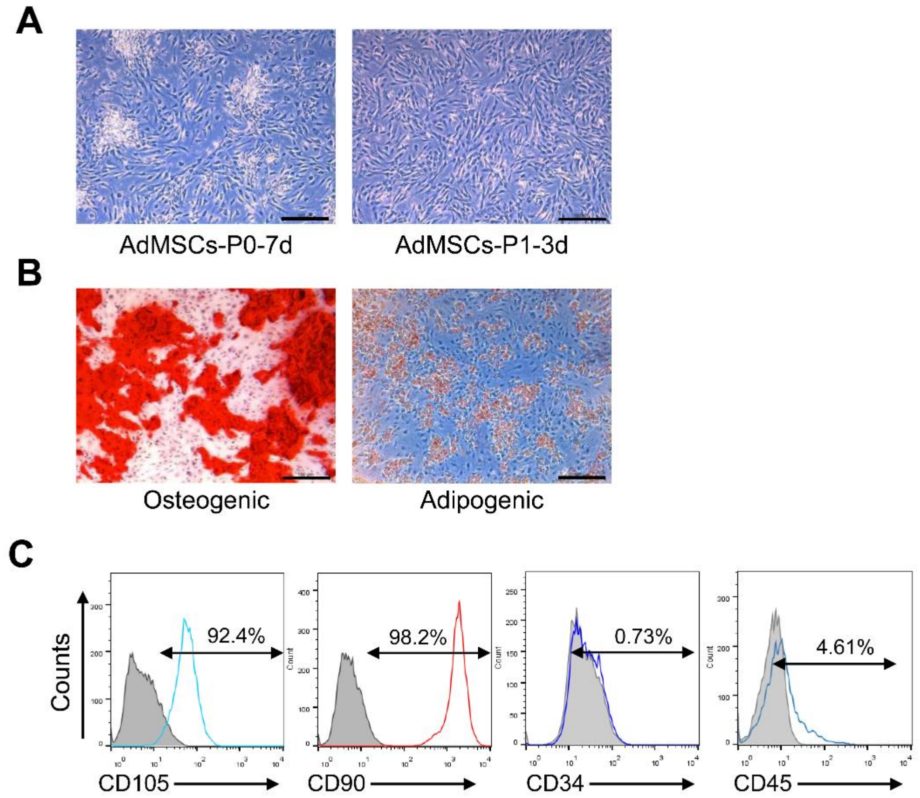

(A) Morphological changes of AdMSCs in rats at different culture time. (B) Under specific differentiation conditions, AdMSCs were differentiated into osteoblasts, adipocytes. Scale bar, 100 μm. (C) Flow cytometry histograms show the positive or negative immunophenotype of cultured AdMSCs. The cells expressed CD105, CD90, CD34 and CD45, known as MSC markers.

FIGURE S2. Observation of the internalization of AdMSC-Exos by MH-S cells after using an endocytosis inhibitor

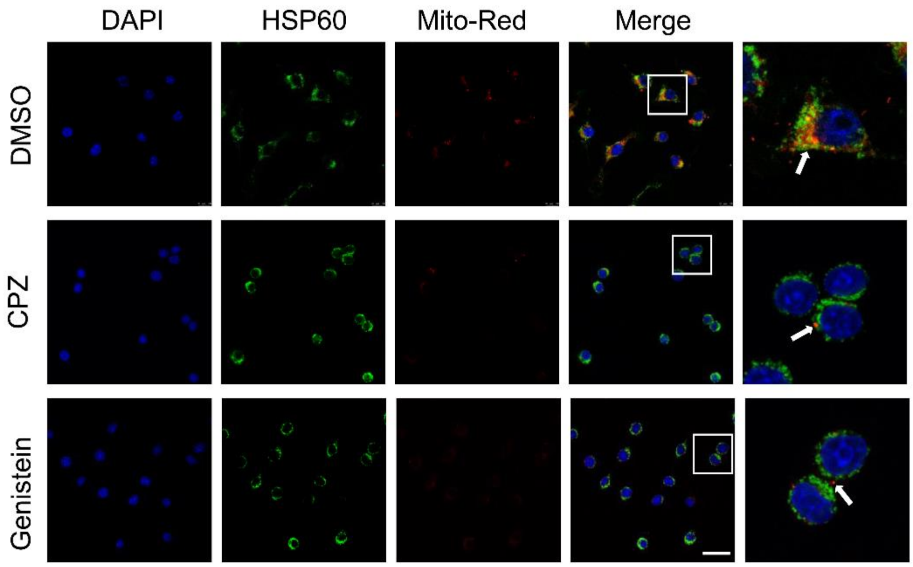

MH-S cells pre-treated with 10 μg/mL chlorpromazine (CPZ) or 200 μM genistein for 30 min were cultured with 10 μg/mL MitoRed-labeled AdMSC-derived exosomes for another 4 h. Internalization of exosomes by MH-S cells was observed using confocal laser scanning microscopy. Scale bar, 25 μm. MitoRed: red, HSP60: green, DAPI: blue.

FIGURE S3. Effect of exosomal-mtDNA and free-mtDNA on sterile inflammation in macrophages

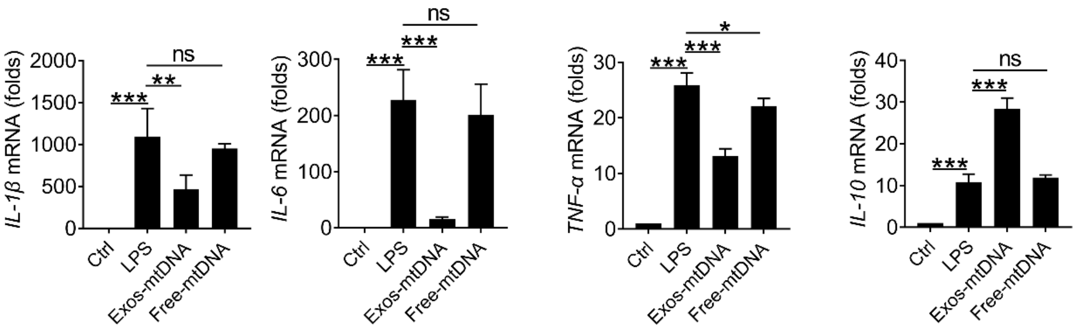

MH-S cells was pretreated with the exosomal-mtDNA isolated from exosomes and the free-mtDNA isolated from AdMSCs 30 min, respectively. Then stimulated with LPS (100 ng/mL) for another 12 h, and the inflammatory response of macrophages was observed by qPCR. All the data are expressed as the mean ± SD; *P < 0.05, **P < 0.01, ***P < 0.001.

FIGURE S4. Effect of exosomes on MH-S cell membrane potential

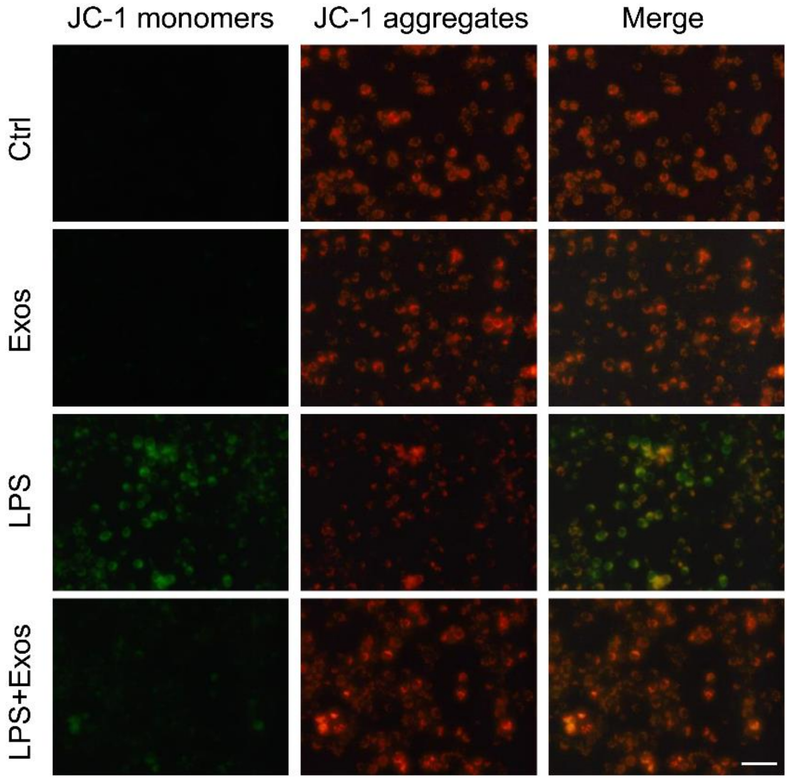

MH-S cells were pretreated with AdMSC-Exos or PBS for 30min, and then subjected to LPS (100 ng/mL) stimulation for 4 h. A JC-1 Staining Kit was used to detect the MMP of MH-S cells, which was observed and visualized by fluorescence microscopy. JC-1 polymer is shown in red and JC-1 monomer is shown in green. Scale bar = 50 μm.

FIGURE S5. AdMSC-exos improved macrophage anti-inflammatory and mitochondrial metabolism tended to replenish damaged mitochondrial components such as NDUFV2

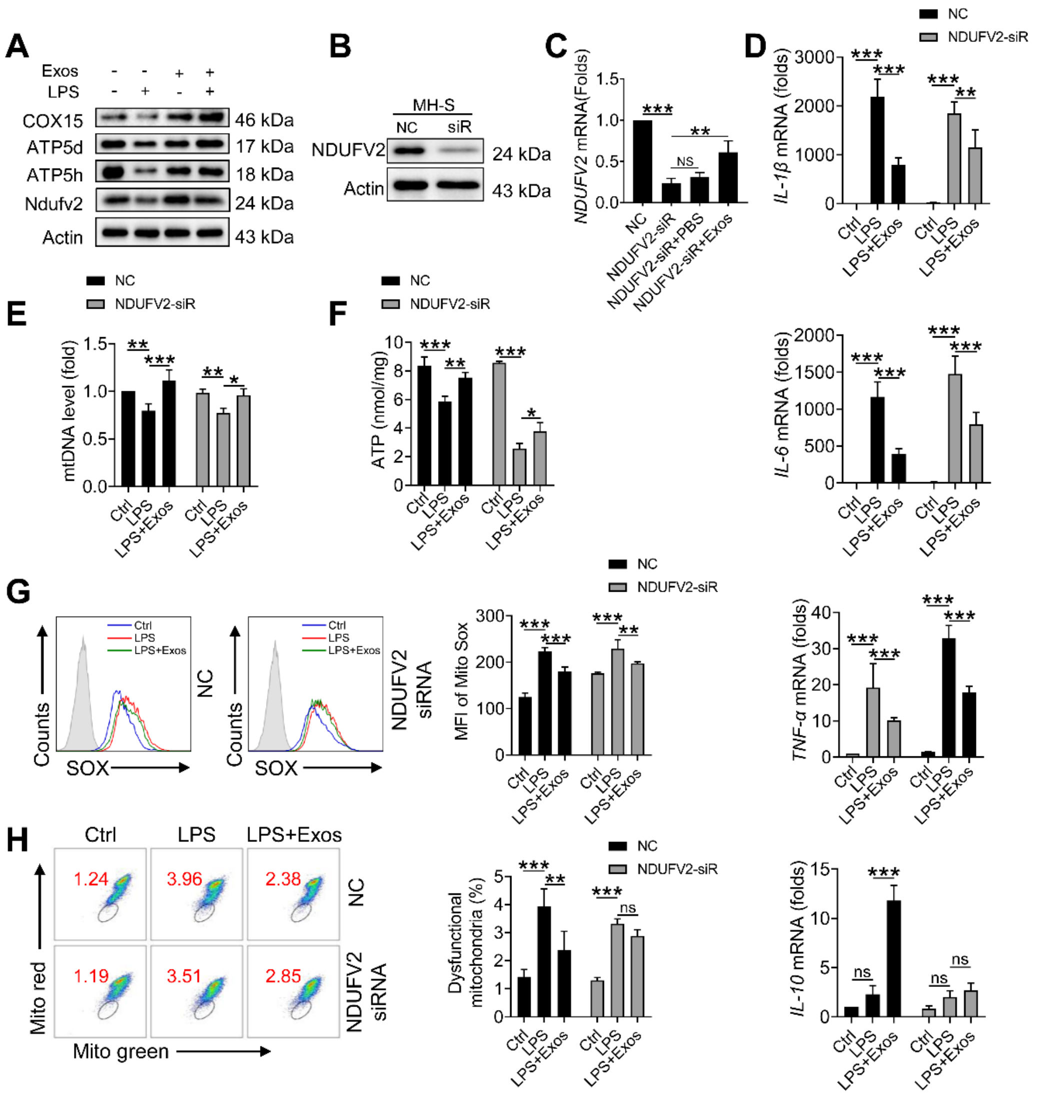

(A) The protein expression levels of mitochondrial respiratory chain-related complexes were detected by western blot. (B) Transfection efficiency of MH-S transfected with siRNA targeted by NDUFV2. (C) Expression of NUDFV2 in MH-S cells by qPCR. (D) Immune inflammatory response of normal MH-S cells and MH-S cells transfected with NDUFV2-targeted siRNA after LPS induction were detected by qPCR. (E, F) Assay of mitochondrial DNA copy number and ATP generation. (G) Flow cytometry and quantification of mitochondrial reactive oxygen species (ROS) levels by staining with Mito Sox. (H) Flow cytometry of mitochondria staining with Mito Tracker Red and Mito Tracker Green. Data represent the mean ± SD of three independent experiments. *P < 0.05, **P < 0.01, ***P < 0.001.

FIGURE S6. Effect of AdMSC-exo on MH-S inflammation and mitochondrial function after knockdown of NDUFV2 in AdMSCs

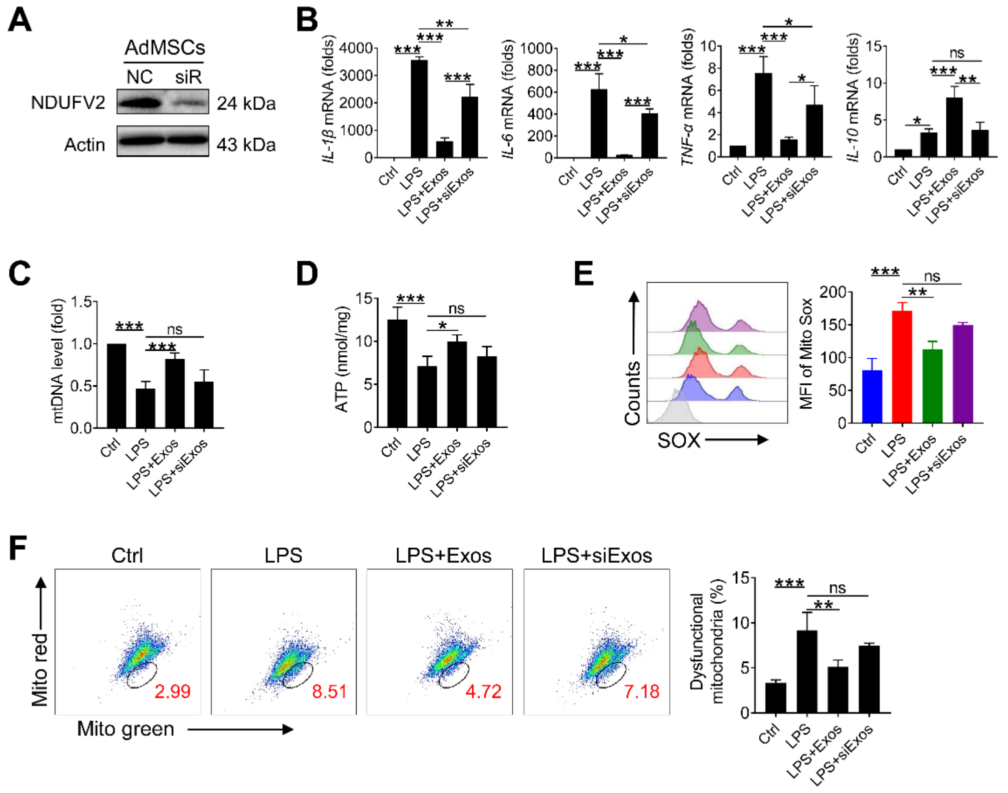

(A) Transfection efficiency of AdMSCs transfected with siRNA targeted by NDUFV2. (B) qPCR assay for inflammation-related molecules as shown in MH-S. (C, D) Assay of mitochondrial DNA copy number and ATP generation in MH-S. (E) Flow cytometry and quantification of mitochondrial reactive oxygen species (ROS) levels by staining with Mito Sox. (F) Flow cytometry of mitochondria staining with Mito Tracker Red and Mito Tracker Green. Data represent the mean ± SD of three independent experiments. $*P < 0.05$, $**P < 0.01$, $***P < 0.001$.

FIGURE S7. The protective effect of AdMSC-exos on lung inflammation during LPS stimulation

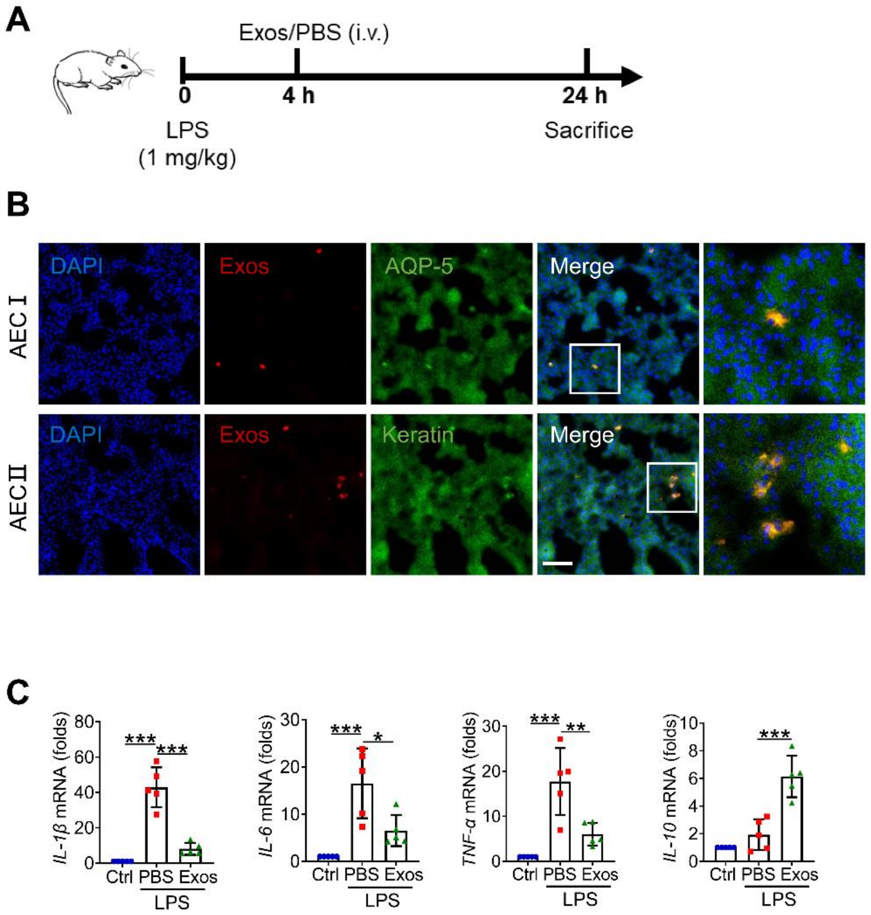

(A) The experimental flowchart of construction of mouse ALI model and AdMSC-exos treatment (n = 5 mice/group). (B) Representative confocal laser scanning microscopy micrographs showing the colocalization of AEC-I marker AQP-5 (green) or AEC-II marker Keratin (green) immunostaining with internalized AdMSC-Exos (red). Scale bar, 10 μm. DAPI: blue. (C) Pro-inflammatory and anti-inflammatory cytokines levels in lung tissue. All the data are expressed as the mean ± SD; *P < 0.05, **P < 0.01, ***P < 0.001.

FIGURE S8. The protective effect of Exos-DNase on lung inflammation was significantly attenuated during LPS stimulation

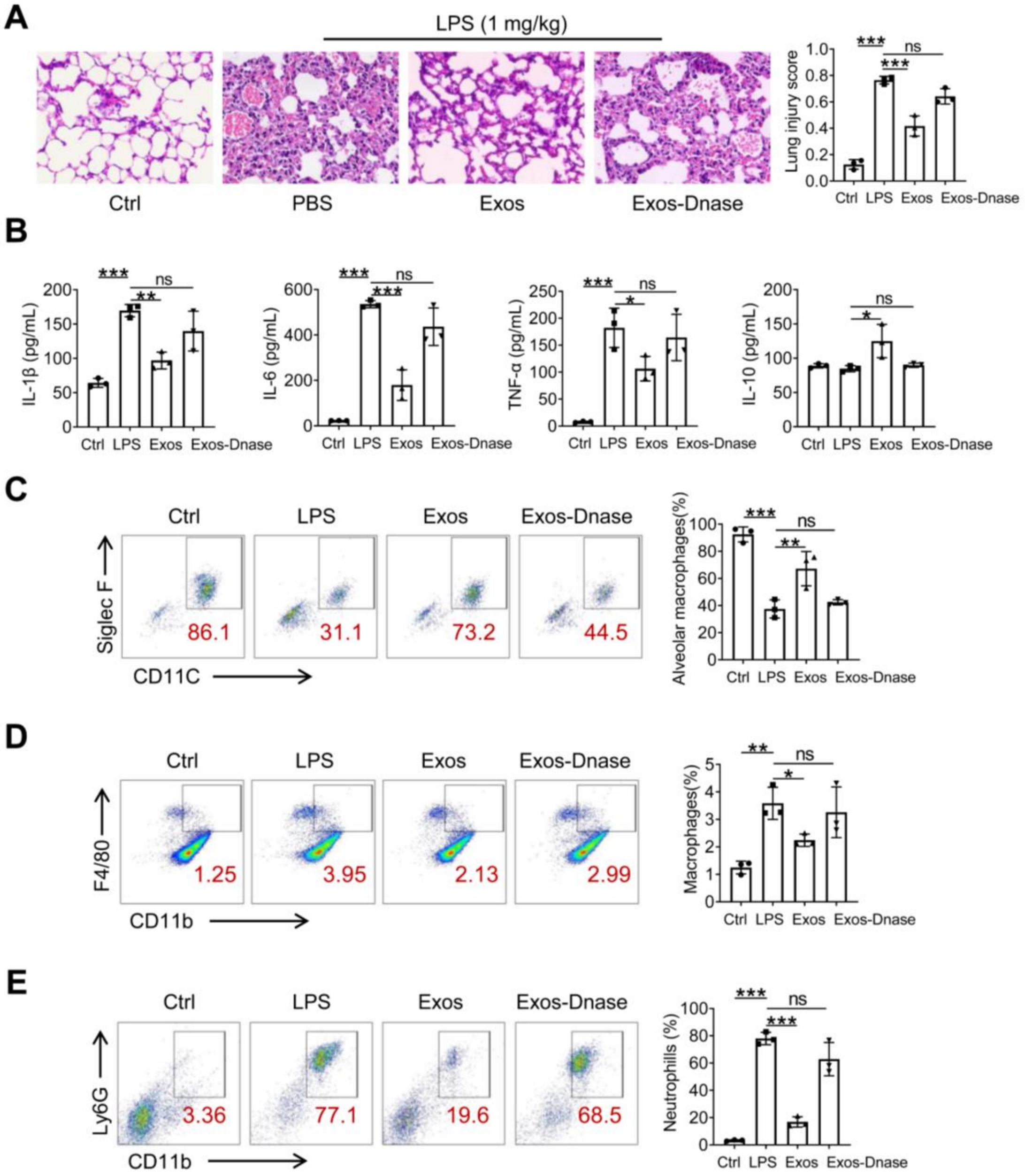

C57BL/6 mice (n = 3 mice/group) were challenged with LPS (1 mg/kg, intratracheally) for 4 h, and then tail vein injected with PBS, Exos (10 μg/mL) or Exos-Dnase (10 μg/mL). 24 h later, mice were sacrificed and subjected to the functional analysis. (A) Representative H&E staining of lung tissues (Scale bar, 50 μm). The histogram shows the lung tissue pathological damage score. (B) Cytokines levels in the BAL fluid by ELISA assay. (C) Flow cytometry analysis of Alveolar macrophages ($CD11c^+$ Siglec F^+) in BAL fluid (Gate on $CD11b^-$ $CD64^+$). (D) Flow cytometry analysis of macrophages ($CD11b^+$ $F4/80^+$) in BAL fluid. (E) Flow cytometry analysis of neutrophils ($CD11b^+$ $Ly6G^+$) in BAL fluid. All the data are expressed as the mean ± SD; *P < 0.05, **P < 0.01, ***P <

0.001.

Figure S9. Schematic diagram of the flow cytometry experimental gate.

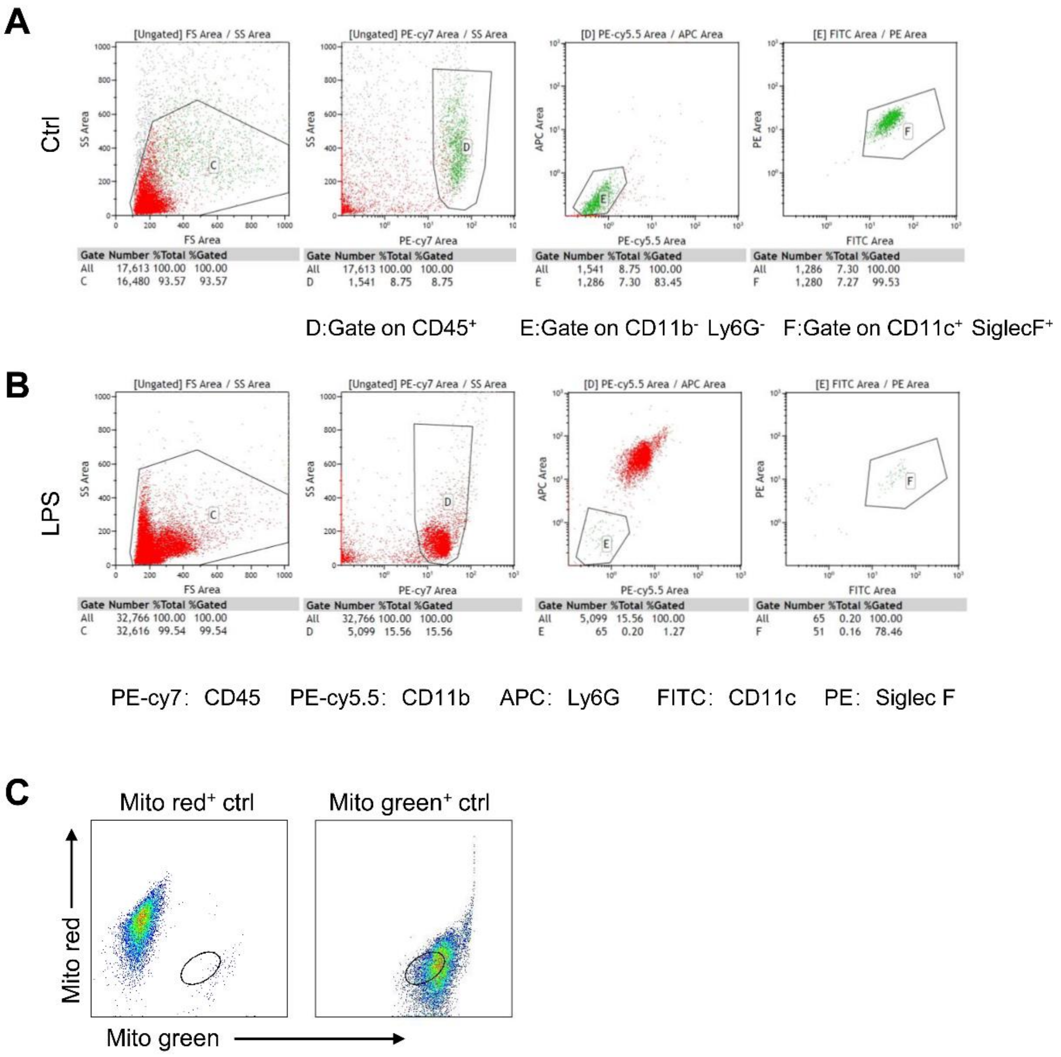

(A, B) Schematic diagram of alveolar macrophage gate in BALF. First, select all immune cells in the alveolar lavage fluid by delineating the CD45-positive cell population. Then select CD11b$^+$ Ly6G$^-$ neutrophils and CD11c$^+$ SiglecF$^+$ alveolar macrophages. (A) The alveolar lavage fluid of mice in the normal control group is dominated by alveolar macrophages, and neutrophils are almost nonexistent. (B) There are a large number of neutrophils in the alveolar lavage fluid of mice with LPS-induced acute lung injury, and the content of alveolar macrophages is significantly reduced. (C) Flow cytometry of mitochondria staining with MitoTracker Red and MitoTracker Green. MH-S respectively stained with MitoTracker Red and MitoTracker Green as controls.

Table 1 Primer sequences

Gene name	Forward primer (5'→3')	Reverse primer (5'→3')
β-actin	CTCATGAAGATCCTGACCGAG	AGTCTAGAGCAACATAGCACAG
IL-6	TTCTTGGGACTGATGCTG	CTGGCTTTGTCTTTCTTGTT
IL-1β	AGGCTCCGAGATGAACAA	AAGGCATTAGAAACAGTCC
TNF-α	TGTCCCTTTCACTCACTGGC	CATCTTTTGGGGGAGTGCCT
iNOS	CCCTTCCGAAGTTTCTGGCAGCAGC	GGCTGTCAGAGCCTCGTGGCTTTG
IL-10	ACAGCCGGGAAGACAATAACT	GCAGCTCTAGGAGCATGTGG
Arg1	CTGGGGATTGGCAAGGTGAT	CGTTGAGTTCCGAAGCAAGC
mtDNA region 1	TGAACGGCTAAACGAGGGTC	AGCTCCATAGGGTCTTCTCGT
mtDNA region 2	CAGTCCCCTCCCTAGGACTT	ACCCTGGTCGGTTTGATGTT
mtDNA region 3	TAATCGCACATGGCCTCACA	GAAGTCCTCGGGCCATGATT
gDNA B2m	AGCAAAGAGGCCTAATTGAAGTC	GAAGTAGCCACAGGGTTGGG
gDNA Tuba1a	TGAGGAGGTTGGTGTGGATTC	TGAGGAGGTTGGTGTGGATTC
ND1	TTCTAATCGCAATGGCATTCCT	AAGGGTTGTAGTAGCCCGTAG
ND5	TTCATCCCTGTAGCATTGTTCG	GTTGGAATAGGTTGTTAGCGGTA
Ndufa4	CTGGAGCAGCACTGTATGTGA	TTGGGACCCAGTTTGTTCCAT
Ndufa11	TCCGCTTACAGCGTCTCAC	AGGCCAAACATCGCTCCAAT
Ndufb3	GAGTTTATGCTGTGCCGCTG	TACTCTGTGAAAGGCTCCGC
Ndufb7	GACCCCGAGAAGATACCCAG	GCACAGTAGTCACGTTGCTG
Ndufb9	ACCGGTACTTTGCTTGCTTG	ATCTCTCGAAGGAAGTGCCC
Ndufb11	GTCCTCCAGGGCTGTAATCG	AAAGTCAGGGTTCTTCGCGT
Ndufv1	TGCTTGTGGCTCCGACTATG	ACAGTTGTGGGGCATCCAAA
Ndufv2	GGAGGAGCCTTATTTGTGCAT	TTTGGGCGAGATCCAGGACT
sdhb	CAGAGTCGGCCTGCAGTTT	ATCCAACACCATAGGTCCGC
sdhd	CTGGTTCCAAGGCTGCATCT	AGCCAGAGAGTAGTCCACCA
Cyc1	ATCGTTCGAGCTAGGCATGG	GCCGGGAAAGTAAGGGTTGA
Uqcr11	GGAACTGGCCAGAAACTGGA	TGCCGTTGATGTAAGGCACC
Uqcrc1	ATGCTGCGTGACATTTGCTC	TAGAAGCGCAGCCAGAACAT
Uqcrq	ATCTCCTACAGCTTGTCGCC	CTGCTCAAACTCCTGGTTGC
Cox5a	TGTCTGTTCCATTCGCTGCT	AACCGTCTACATGCTCGCAA
Cox5b	GCTTCAAGGTTACTTCGCGG	ATGGGTCCAGTCCCTTCTGT
Cox6a1	CAACGTGTTCCTCAAGTCGC	CTTCATAGCCGGTCGGAAGT
Cox6b1	AGAACTACAAAACTGCCCCCT	TTCTCACAGCGGTGGAAGTC
Cox7c	GAGTATCCGGAGGTTCACGAC	ACCGCCACTTGTTTTCCACT
Cox8a	CAGGTCCACTCGAAGCCG	CAGGCAGAAGACAACACACG
Cox15	GCGTCCGGCAACGGT	TGATGGTGCTGTACTGTCCT
ATP5d	TACGCTGACTGGAGCCTTTG	GTCCAGCATGTCCAGTGTCA
ATP5e	TCAGCTACATCCGGTTTTCCC	TTTTATGCTGCTGCCCGAAG
ATP5g2	ATGTACGCCTGCTCCAAGTT	CTGTGGTCGCTTCAACTCCA
ATP6v1	ACATCGCAGAGATGGTTCGG	CTTTGGCTGCATCGTAGGGA
ATP6v0c	GTCCCGTTGTCCTAGCTCGC	TCCTAGAAGCTGGGTGCAGAA
ATP5h	TGGAATGAGACCTTCCACGC	GCACAGGAATCTTCAGGGCA
ATP5k	TACCTAAAACCCCGGGCAGA	CATCTTGAGCTTCCGCCAGT

Table 2 Alignment of mouse and human mitochondrial DNA sequences

```
HND1 ----------------------------------------------------------------------------------------------------
MND1 GAAGCAACCTTAATCCCAACACTTATTATTATTACCCGATGAGGGAACCAAACTGAACGCCTAAACGCAGGGATTTATTTCCTATTTTATACCCTAATCG

HND1 ----------------------------------------------------------------------------------------------------
MND1 GTTCTATTCCACTGCTAATTGCCCTCATCTTAATCCAAAACCATGTAGGAACCCTAAACCTCATAATTTTATCATTCACAACACACACCTTAGACGCTTC

                           HND1 F
                           ------------------------->
HND1 ----------------------TTCTAAT-CGCAATGGCATTCCTAATGCTTACCGAACGAAAAATTCTAGGCTATATACAACTACGCAAAGGCCCCAACG
MND1 ATGATCTAACAACTTACTATGGTTGGCATGCATAATAGCATTTCTTAT---TAAAATACCATTATATGGAGTTCACCTATGACTAC-CAAAAGCCC--ATG

                       HND1 R
             <------------------
HND1 TTGTAGGCCC--CTACGGGCTACTACAACCCTT--------------------------------------------------------------------
MND1 TTGAAGCTCCAATTGCTGGGT-CAATAATTCTAGCAGCTATTCTTCTAAAATTAGGTAGTTACGGAATAATTCGCATCTCCATTATTCTAGACCCACTAAC

HND1 -----------------------------
MND1 AAAATATATAGCATACCCCTTCATCCTTC


      HND5 F
      ------------------------->
HND5 TTCATCCCTGTAGCATTGTTCGTTACATGGTCCATCATAGAATTCTCACTGTGATATATAAACTCAGACCCAAACATTAATCAGTTCTTCAAATATCTACT
MND5 -----------------------------------------------CCTGGCAGACGAACAAGACATCCGAAAAATAGGAAACATCA-CAAAAATCATAC

                                       HND5 R
                               <------------------
HND5 CATCTTCCTAATTAC-CATACTAATCTTAGTTACCGCTAACAACCTATTCCAAC
MND5 CATTCACATCATCATGCCTAGTAATCGGAAGCCTCG------------------
```

Table 3 Antibody type and applications

Antibody Name	Company	Catalog Number	Reactivity	Dilution
PE-conjugated CD34 Antibody	BD	560941	H	1 test/10^6 cells
PE-conjugated CD45 Antibody	BD	560975	H	1 test/10^6 cells
Pecy5.5-conjugated CD90 Antibody	BD	561557	H	1 test/10^6 cells
FITC-conjugated CD105 Antibody	BD	561443	H	1 test/10^6 cells
PE-conjugated MHC-II Antibody	ebioscience	12-5321-82	M	1 test/10^6 cells
APC-conjugated CD206 Antibody	ebioscience	17-2061-82	M	1 test/10^6 cells
Pecy7-conjugated CD45 Antibody	MultiSciences	AM04510-100	M	1 test/10^6 cells
Pecy5.5-conjugated CD11b Antibody	ebioscience	45-0112-80	M	1 test/10^6 cells
FITC-conjugated CD11c Antibody	ebioscience	11-0114-82	M	1 test/10^6 cells
PE-conjugated F4/80 Antibody	BD	T45-2342	M	1 test/10^6 cells
APC-conjugated Ly6G Antibody	MultiSciences	AM0L605-100	M	1 test/10^6 cells
PE-conjugated Siglec F Antibody	ebioscience	12-1702-80	M	1 test/10^6 cells
β-Actin Antibody	CST	3700	M,H,R	WB 1:2000
GAPDH Antibody	CST	5174	M,H,R	WB 1:2000
CD9 Antibody	Abcam	Ab92726	H	WB 1:1000
CD63 Antibody	Abcam	Ab216130	H,M	WB 1:1000
TSG101 Antibody	Abcam	Ab83	H,M	WB 1:1000
VDAC Antibody	Abcam	Ab154856	M,H,R	WB 1:1000
TOM20 Antibody	Santa Cruz	sc-17764	M,H,R	WB 1:1000
TFAM Antibody	Absin	abs136216	H,R	WB 1:1000
CALR	Santa Cruz	sc-373863	M,H,R	WB 1:1000
LAMP1	Abcam	Ab25245	H	WB 1:1000
F4/80	Abcam	Ab100790	M,H	IF 1:200
AQP-5	Santa Cruz	sc-514022	M,H,R	IF 1:200
Keratin	Proteintech	10712-1-AP	M,H	IF 1:200
Cox15 Antibody	Proteintech	11441-1-AP	M,H,R	WB 1:1000
NDUFV2 Antibody	Proteintech	15301-1-AP	M,H,R	WB 1:1000
ATP5D Antibody	Proteintech	14893-1-AP	M,H,R	WB 1:1000
ATP5H Antibody	Proteintech	17589-1-AP	M,H,R	WB 1:1000
PGC1α Antibody	Proteintech	66369-1-lg	M,H,R	WB 1:1000
Sirt1 Antibody	CST	8469	M,H,R	WB 1:1000
p65 Antibody	CST	8242	M,H,R	WB 1:1000
p-p65 Antibody	CST	13346	M,H,R	WB 1:1000
IKKα Antibody	CST	61294	M,H,R	WB 1:1000
p-IKKα Antibody	CST	2697	M,H,R	WB 1:1000
Iκbα Antibody	CST	4812	M,H,R	WB 1:1000
p-Iκbα Antibody	CST	2859	M,H,R	WB 1:1000
JNK Antibody	CST	9252	M,H,R	WB 1:1000
p-JNK Antibody	CST	9255	M,H,R	WB 1:1000
ERK Antibody	CST	4695	M,H,R	WB 1:1000
p-ERK Antibody	CST	4370	M,H,R	WB 1:1000

p38 Antibody	CST	8690	M,H,R	WB 1:1000
p-p38 Antibody	CST	9216	M,H,R	WB 1:1000
HSP60 Antibody	Proteintech	66041-1-Ig	M,H,R	IF 1:200
Draq5 Antibody	Abcam	Ab108410	M,H,R	IF 1:1000
DAPI Antibody	Beyotime	C1002	M,H,R	IF 1:1000
